# A Simple Auxin Transcriptional Response System Regulates Multiple Morphogenetic Processes in the Liverwort *Marchantia polymorpha*

**DOI:** 10.1371/journal.pgen.1005207

**Published:** 2015-05-28

**Authors:** Eduardo Flores-Sandoval, D. Magnus Eklund, John L. Bowman

**Affiliations:** 1 School of Biological Sciences, Monash University, Melbourne, Victoria, Australia; 2 Department of Plant Biology, University of California, Davis, Davis, California, United States of America; Harvard University, UNITED STATES

## Abstract

In land plants comparative genomics has revealed that members of basal lineages share a common set of transcription factors with the derived flowering plants, despite sharing few homologous structures. The plant hormone auxin has been implicated in many facets of development in both basal and derived lineages of land plants. We functionally characterized the auxin transcriptional response machinery in the liverwort *Marchantia polymorpha*, a member of the basal lineage of extant land plants. All components known from flowering plant systems are present in *M*. *polymorpha*, but they exist as single orthologs: a single *MpTOPLESS* (*TPL*) corepressor, a single *MpTRANSPORT INHIBITOR RESPONSE 1* auxin receptor, single orthologs of each class of *AUXIN RESPONSE FACTOR* (*ARF; MpARF1*, *MpARF2*, *MpARF3*), and a single negative regulator *AUXIN/INDOLE-3-ACETIC ACID* (*MpIAA*). Phylogenetic analyses suggest this simple system is the ancestral condition for land plants. We experimentally demonstrate that these genes act in an auxin response pathway — chimeric fusions of the MpTPL corepressor with heterodimerization domains of MpARF1, MpARF2, or their negative regulator, MpIAA, generate auxin insensitive plants that lack the capacity to pattern and transition into mature stages of development. Our results indicate auxin mediated transcriptional regulation acts as a facilitator of branching, differentiation and growth, rather than acting to determine or specify tissues during the haploid stage of the *M*. *polymorpha* life cycle. We hypothesize that the ancestral role of auxin is to modulate a balance of differentiated and pluri- or totipotent cell states, whose fates are determined by interactions with combinations of unrelated transcription factors.

## Author Summary

In flowering plants it is hypothesized the complexity and the robustness of the auxin transcriptional response could be generated by a large number of paralogs encoding components of this signaling network. Yet, it is not known whether alternative embryophyte body plans can be patterned with a simpler auxin transcriptional machinery. We demonstrate that in the liverwort *Marchantia polymorpha*, the type rather than the number of components of the auxin transcriptional response pathway are sufficient to pattern a complex three-dimensional gametophyte with multiple tissue and cell types. In *M*. *polymorpha*, mutations in components of the auxin signaling pathway cause dramatic pleiotropic effects despite having a single class A activating *AUXIN RESPONSE FACTOR* (*MpARF1*). This supports the hypothesis that auxin is context dependent and that it facilitates rather than specifies particular developmental processes. We show that auxin signaling is not necessary for survival but it is required to pattern the transition from two-dimensional to three-dimensional growth. This suggests that the evolution of the auxin transcriptional response was critical for the evolution of developmental complexity in land plants.

## Introduction

Genetic and physiological studies have revealed that the phytohormone auxin, indole-3-acetic acid (IAA), controls central aspects of plant development in both haploid and diploid generations of plant species. Auxin triggers growth and tissue differentiation in places where it accumulates, perhaps acting as a morphogenetic trigger [1]. In the flowering plant *Arabidopsis thaliana*, a balance between auxin synthesis, transport, conjugation/de-conjugation and degradation regulates the spatial distribution of auxin, leading to activation of transcriptional responses that influence a variety of morphological and physiological processes [2,3,4,5,6,7,8].

The auxin transcriptional response in its simplest outline is as follows. In the absence of auxin, AUXIN/INDOLE-3-ACETIC ACID (AUX/IAA) repressors heterodimerize with AUXIN RESPONSE FACTOR (ARF) transcription factors (via two conserved domains, D34) and recruit TOPLESS (TPL) corepressors, thus preventing ARFs from regulating target genes [9,10,11,12,13]. In the presence of auxin, the AUX/IAA proteins are targeted for degradation by an SCF E3 ubiquitin ligase (TRANSPORT INHIBITOR RESPONSE1 [TIR1]/AUXIN SIGNALING F-BOX [AFB]), via auxin dependent interactions between AUX/IAA and TIR1 proteins [14,15,16,17]. Phylogenetic analyses of *ARF* evolution in land plants identifies three ARF classes: class A (orthologues of *Arabidopsis ARF5-8*), class B (orthologues of *Arabidopsis ARF1-4*, *ARF9*) and class C (orthologues of *Arabidopsis ARF10*, *ARF16*, *ARF17*) [18,19,20]. However, not all classes of ARFs are equal, with class A acting primarily as activators, and classes B and C as repressors [19,20]. Since auxin application predominantly activates gene expression, it has been argued that class B and C ARFs may act to repress common target genes activated by class A ARFs since all classes can potentially bind the same auxin responsive element (AuxRE) in promoters of auxin responsive genes [19,20,21]. Among the direct targets of ARFs are genes encoding AUX/IAA proteins, establishing a negative feedback loop [22,23]. Studies in *Arabidopsis* have suggested that activator ARFs interact with AUX/IAAs and that AUX/IAAs may also homodimerize, but that repressor ARFs do not interact efficiently with the AUX/IAAs, leaving most class B and C repressors outside this protein network [10]. These results imply that many ARF repressors regulate gene expression independently of auxin, as TIR1/AFB mediated degradation of AUX/IAAs would not affect their activity. However, all ARF proteins potentially compete for the same targets as they recognize similar AuxREs [11,21]. Thus, a current view is that transcriptional activation by class A ARFs is negatively regulated in an auxin dependent manner by AUX/IAAs and an auxin independent manner by class B and C ARFs.

That 23 ARF, 29 AUX/IAA, 6 TIR1/AFB, and 5 TPL paralogs are encoded in the *Arabidopsis* genome has prompted speculation that interactions differing in affinity among paralogs could influence auxin sensitivity in different cells [10,24]. Key features of the auxin signaling pathway are mechanistically similar in the moss *Physcomitrella patens* suggesting conservation of the auxin transcriptional response throughout land plants [25]. Given the complexity arising from multiple paralogs encoding each of the components of the auxin transcriptional response pathway in both flowering plants and *P*. *patens* [18], we sought to establish a model system where such complexity is reduced. It has been speculated that an increase in complexity of the auxin genetic toolkit (including genes for metabolism, synthesis, transport and signaling) is correlated with an increased complexity in land plant architecture, from bryophytes to flowering plants [26]. Although there is no universal agreement on the phylogenetic relationships of extant land plant clades, it is generally accepted that liverworts diverged earlier than other land plants based on morphology, fossil evidence, and some molecular analyses [27,28,29]. Liverworts in general have experienced a low rate of chromosomal evolution, with little evidence of widespread polyploidy, and molecular evolution within the Marchantiopsida is reported to be slow, compared with more derived liverwort lineages [30,31]. Given these evolutionary attributes, we reasoned that *Marchantia polymorpha* could possess a simplified version of the auxin response system. Furthermore, as *M*. *polymorpha* has been used as a model system for nearly two centuries to investigate both universal and specific biological processes, an extraordinary body of literature is available to inform our studies.

In *M*. *polymorpha*, the haploid spore produces a short protonemal filament only a few cells long [32,33,34]. The brief protonemal stage is followed by the formation of a globular sporeling that subsequently develops a marginal row of cells in its apical (distal) region, a central cell of which will form the apical cell of the gametophytic shoot meristem [33,34,35]. The marginal row of cells is conspicuous and forms the distal growing tip, and represents the transition from a sporeling to a 'prothallus'—a flattened unistratose heart shaped plant that grows by means of an apical cell with two alternate cutting faces [33,35]. Subsequently, a change in the wedge-shaped apical cell such that divisions occur along four planes, rather than two, transforms the prothallus into a thallus. Accompanying this transition is the establishment of dorsiventrality, with the production of specialized cell types both dorsally and ventrally [36]. *M*. *polymorpha* can also reproduce asexually via small, flattened lecticular gemmae [32,33,37,38]. Mature gemmae are dormant and lack dorsiventral polarity while inside dorsal gemmae cups, but acquire environmentally induced polarity when displaced from the gemmae cup. Individual gemmae are clonal and derived from a single cell of the parental thallus [32,33,37,39].

The effects of exogenous auxin on *M*. *polymorpha* gemmaling growth have been reported in previous studies. Some generalizations of the effects of exogenous auxin in young gemmalings can be made as follows: in low concentrations auxin increases the size of the thallus [40], breaks gemmae dormancy [41,42], produces formation of additional meristems in dormant gemmae [43], and confers apical dominance [44]. At higher concentrations auxin promotes the formation of numerous rhizoids on the dorsal side of the gemmae, a dorsiventral patterning defect, and further increases in concentration results in formation of a callus-like tissue with limited growth [40,41,42,43,44,45,46,47,48,49,50,51,52,53,54]. In transgenic *M*. *polymorpha*, an auxin-responsive regulatory sequence derived from soybean, the *GRETCHEN HAGEN 3* (*GH3*) promoter, _*pro*_ *GmGH3*, responds to exogenous auxin in a dose dependent manner [40], and consistent with previous pharmacological experiments, sites of _*pro*_ *GmGH3* expression in un-induced lines partially overlap with sites of auxin action, including incipient rhizoids and the bases of gemmae cups.

In the present work, we show that the liverwort *M*. *polymorpha* possesses the simplest auxin genetic toolkit so far found in any land plant. Dissection of the auxin response system of *M*. *polymorpha*, points to auxin being a facilitator rather than a determiner of cell fate, with auxin also being required for major developmental transitions during gametophyte development.

## Results

### Auxin influences most developmental stages of *M*. *polymorpha* gametophyte development

To address the role of auxin in the *M*. *polymorpha* gametophyte, young undifferentiated gemmae and young thalli with established dorsiventral polarity were grown in the presence of the auxin analogues naphthalene-1-acetic acid (NAA) and 2,4-dichlorophenoxyactetic acid (2,4-D).

**Fig 1:**
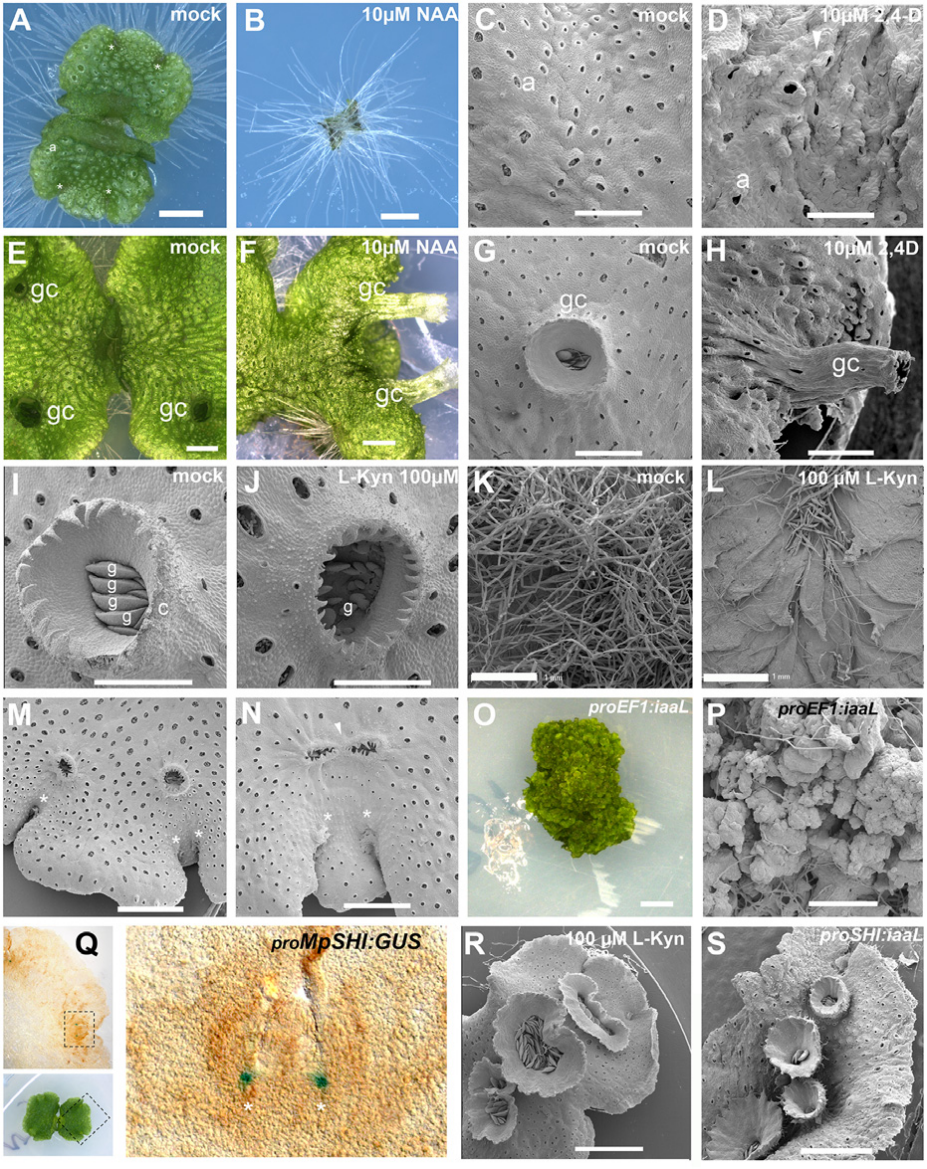
Developmental effects of auxin in the *M*. *polymorpha* thallus. **(A)** 10-day-old wild-type gemmaling with four apical notches and well developed air pores. **(B)** 10-day-old wild-type gemmae grown on 10 μM NAA. **(C)** Dorsal epidermis of 15-day-old wild-type thallus. **(D)** Dorsal epidermis of 15-day-old wild-type thallus, transferred on day 6 to 10 μM 2,4-D with abnormal air pores. **(E)** 23-day-old wild-type thallus with three gemmae cups. **(F)** 23-day-old wild-type thallus transferred at day 7 to 10 μM NAA with elongated gemmae cup. **(G)** Wild-type mature gemmae cup. **(H)** Wild-type mature gemmae cup from 17-day-old plant, transferred at day 12 to 10 μM 2,4-D. **(I)** Wild-type mature gemmae cup with gemmae inside. **(J)** Wild-type mature gemmae cup from a plant grown on 100 μM L-Kyn. **(K)** Ventral side of wild-type thallus grown on mock media. **(L)** Ventral side of wild-type thallus grown on 100 μM L-Kyn; a decrease in rhizoid number exposes ventral scales. **(M)** Mature thallus showing a thallus lobe separating two recently bifurcated apical notches. **(N)** Mature thallus of plants transferred on day 7 to 100 μM L-Kyn, showing a smaller lobe spacing apical notches and fused gemmae cup primordia (arrowhead). **(O)** Strong _*pro*_ *EF1*:*iaaL* lines are composed of an undifferentiated mass of cells that fail to establish a clear dorsiventrality. **(P)** Close up view of _*pro*_ *EF1*:*iaaL* line shows a mass of undifferentiated cells although rhizoids are able to differentiate. **(Q)** Expression pattern of _*pro*_ *MpSHI* in mature thallus with staining in the vicinity of the apical cell. **(R)** Different degrees of gemmae cup fusion observed in 100 μM L-Kyn treated plants. **(S)**_*pro*_ *MpSHI*:*iaaL* plants show an irregular zig-zag arrangement of gemmae cups that are in close proximity to each other. Scale bars in A, B, E, F, G, H, J, K, L, O, R and S = 1 mm; C, D and P = 0.6 mm; I = 0.5 mm; M = 1.2 mm; N = 0.75 mm; Q = 0.2 mm. Asterisk, apical notch; a, air pores; gc, gemma cups; g, gemmae.

Auxin application to gemmae and gemmalings grown up to four days old on unsupplemented media prior to auxin treatment triggered ectopic rhizoid formation on the dorsal surface of the thallus (Fig 1A and 1B). Normal dorsal structures including air chambers, air pores, and gemmae cups did not develop. However, plants were not entirely ventralized since scales, which are ventral structures, did not develop dorsally. Thalli also failed to develop a normal branching pattern and lacked lateral growth.

Application of auxin to gemmalings with established dorsiventral polarity, those grown for six and sixteen days on unsupplemented media prior to auxin treatment, resulted in two classes of aberrant phenotype. In the first class, occurring primarily in plants with auxin treatment after one week, the thallus developed excessive rhizoids, although they were spatially restricted to the original central zones of the gemmalings and to the ventral side of the mature thallus (S1A and S1B Fig)—locations where rhizoids arise in untreated plants. Thus, by day six of gemmaling development dorsal tissues have lost the capacity to produce rhizoids in response to auxin (S1B Fig). However, typical dorsal structures of the thallus were affected, with air pores often either partially formed or unopened (Fig 1C and 1D, S1B and S1C Fig), air chambers structure asymmetric and disorganized, and an undulating dorsal epidermal surface with conspicuously protruding air pores (Fig 1D). Additionally, plants subject to this treatment were unable to form any gemmae cups (S1A–S1C Fig).

In the second, milder class, primarily occurring in plants with auxin treatment after two weeks, plants exhibited abnormal tubular elongation of gemmae cups that had been initiated prior to auxin treatment (Fig 1E–1H and S1D Fig). The gemmae cups exhibited a positive gravitropic response in contrast to their normal negative graviotropism [55]. While previously formed gemmae cups continued to develop, no new cups were formed. Air chambers were asymmetric, spatially disorganized, and protruding upwards as in the first class (Fig 1F–1H). The growing thallus margins were bent downwards (Fig 1F and S1D Fig), an epinastic growth pattern reminiscent of epinastic angiosperm leaf growth in response to high levels of auxin [56].

### Lack of auxin affects gemmae cup formation and thallus branching

As a complementary approach we examined the phenotypic consequences of reducing endogenous auxin levels. We first tested the effects of the auxin biosynthesis inhibitor L-Kynurenine (L-Kyn) on gemmaling development [57]. In *Arabidopsis* L-Kyn acts as an alternative substrate and competitive inhibitor for the *TRYPTOPHAN AMINOTRANSFERASE OF ARABIDOPSIS1* (*TAA1*) and *TRYPTOPHAN AMINOTRANSFERASE RELATED1*, *2* (*TAR1*, *2*) enzymes, inhibiting the conversion of tryptophan (trp) to indole-3-pyruvic acid (IPA), a key step in auxin biosynthesis [4,57,58]. *Arabidopsis* plants grown in the presence of 100 μM L-Kyn phenocopy *wei8 tar2* double mutants (the *weak ethylene insensitive 8* mutant is a null loss-of-function allele of *TAA1*) [57,59].

Wild-type gemmae grown on 100 to 250 μM L-Kyn were unable to produce normal gemmae cups, with gemmae cup rims failing to fully develop (Fig 1I and 1J). Growth of gemmae was slower as compared to wild type (S1E and S1F Fig), with smaller propagules forming inside the cup (Fig 1I and 1J). Decreased rhizoid production was also observed but was not measured quantitatively (Fig 1K and 1L).

During vegetative growth, a gemmae cup is usually initiated following each branching event [36] and only a single cup is formed per branching event (S2 Fig). Immediately after branching a growth phase separates the two apical notches creating a mid-lobe prior to a new round of branching and gemmae cup formation. Application of L-Kyn to established seven-day-old thalli resulted in gemmae cups derived from successive branching events fusing into a single structure (Fig 1M and 1N). The extent of fusion varied, with the most extreme fusion resulting in single oval gemmae cups (e.g. Fig 1N and 1R). Therefore, inhibiting auxin synthesis reduces the rate of branching and the extent of growth separating branches.

In order to further deplete free auxin levels in the thallus, we created transgenic plants overexpressing a heterologous bacterial auxin conjugation enzyme iaaL [60,61,62] under the control of the *EF1-*α promoter (*proEF1*), which drives high levels of expression in the meristematic regions and throughout the thallus [63]. _*pro*_ *EF1*:*iaaL* plants exhibited disruptions to patterning and growth, with thallus growth severely stunted, despite evidence of continuing cell division (Fig 1O and 1P). Except for a few cells developing as rhizoids, most cells in _*pro*_ *EF1*:*iaaL* plants do not acquire a particular fate, but instead resemble cells present in early stages of sporeling development.

To manipulate free auxin levels specifically in the meristematic region we utilized a 3.2kb promoter fragment of the single *M*. *polymorpha* orthologue of the *SHORT INTERNODES/STYLISH* (*SHI/STY*) gene family [64,65,66]. Under normal growth conditions, _*pro*_ *MpSHI*:*GUS* activity was detected mainly in the apical notch (meristematic region) during all stages of development (Fig 1Q). Most _*pro*_ *MpSHI*:*iaaL* transformants exhibited weaker phenotypes than _*pro*_ *EF1*:*iaaL* and were able to form a narrow thallus with compromised lamina expansion and a reduced rate of branching (Fig 1S, S1G and S1H Fig). Gemmae cups sometimes developed to the side of the midline, and/or close together, suggesting an irregular zig-zag formation and a degree of fusion (Fig 1S), resembling L-Kyn treatments (Fig 1R). We thus conclude that auxin influences the growth and differentiation of multiple tissues and processes in different developmental stages of the *M*. *polymorpha* gametophyte.

### Auxin transcriptional response genes are conserved in *M*. *polymorpha*

Using PCR based methods and genomic sequences provided by the Joint Genome Institute, we identified single *TPL* (*MpTP*L, genbank accession number KP877967), *AUX/IAA* (*MpIAA*, KP877968), and *TIR1/AFB* (*MpTIR*) orthologues in *M*. *polymorpha*, as well as three ARF paralogs (*MpARF1*, KP877969; *MpARF2*, KP877970; *MpARF3*, KP877971; S13 Fig).

Analysis of the *MpTPL* sequence revealed highly conserved LisH, CTLH and WD40 repeat domains, characteristic of this gene family (amino acid sequence identity between *MpTPL* and *Arabidopsis TPL* is 68%). *MpIAA* harbors a conserved EAR domain (D1), as well as a proteolysis domain (D2); D1 allows AUX/IAA proteins to interact with the TPL CTLH domain, and D2 domain has been shown to facilitate interactions between AUX/IAA proteins and TIR1/AFB [15]. Both MpIAA and all three MpARFs possess conserved protein-protein interaction domains (D34; S3 Fig and S1 Table). In an accompanying paper, Kato et al [67] describe the extent of MpARF-MpIAA interactions in *M*. *polymorpha* using yeast two-hybrid (Y2H) and biomolecular fluorescence complementation (BiFC).

Bayesian phylogenetic analyses were performed to elucidate the evolution of the TPL, AUX/IAA, and ARF gene families using sequences from *M*. *polymorpha*, *P*. *patens*, *Selaginella moellendorffii*, *Pseudotsuga menziesii*, *Amborella tricopoda*, *Solanum lycopersicum* and *Arabidopsis thaliana*. The TPL phylogeny suggests that the common ancestor of land plants had a single TPL gene, with the *M*. *polymorpha* genome retaining this ancestral feature. Our analysis suggests a gene duplication in the lineage leading to the origin of tracheophytes (maximum support), creating two classes of TPL genes (Class A and B). This topology implies a loss of class A genes in *S*. *moellendorffii* (S4 Fig) and further sequence sampling may clarify if this is a trend in other lycophytes.

Phylogenetic analysis of the AUX/IAA family also suggests a single gene in the embryophyte ancestor, rendering *M*. *polymorpha* ancestral for AUX/IAA gene copy number. Our phylogenetic tree indicates that angiosperms and gymnosperms also have two types of AUX/IAAs, class A (similar to the *Arabidopsis IAA1-7*, *IAA9*, *IAA14-17*, *IAA19* and *IAA27*) and class B AUX/IAAs (including *IAA10-13*, *IAA18*, *IAA26*, *IAA28* and *IAA29*), suggesting a gene duplication in the common ancestor of seed plants (S5 Fig).

We performed Bayesian analysis of land plant ARF genes, and as noted in previous analyses, our phylogenetic analysis results in a tree with three distinct and well-supported ARF clades (A, B, C) (S6 Fig). *M*. *polymorpha* possesses a single homolog of each of these classes, *MpARF1* (A), *MpARF2* (B), and *MpARF3* (C).

### Loss of *MpIAA* results in auxin hypersensitivity

**Fig 2:**
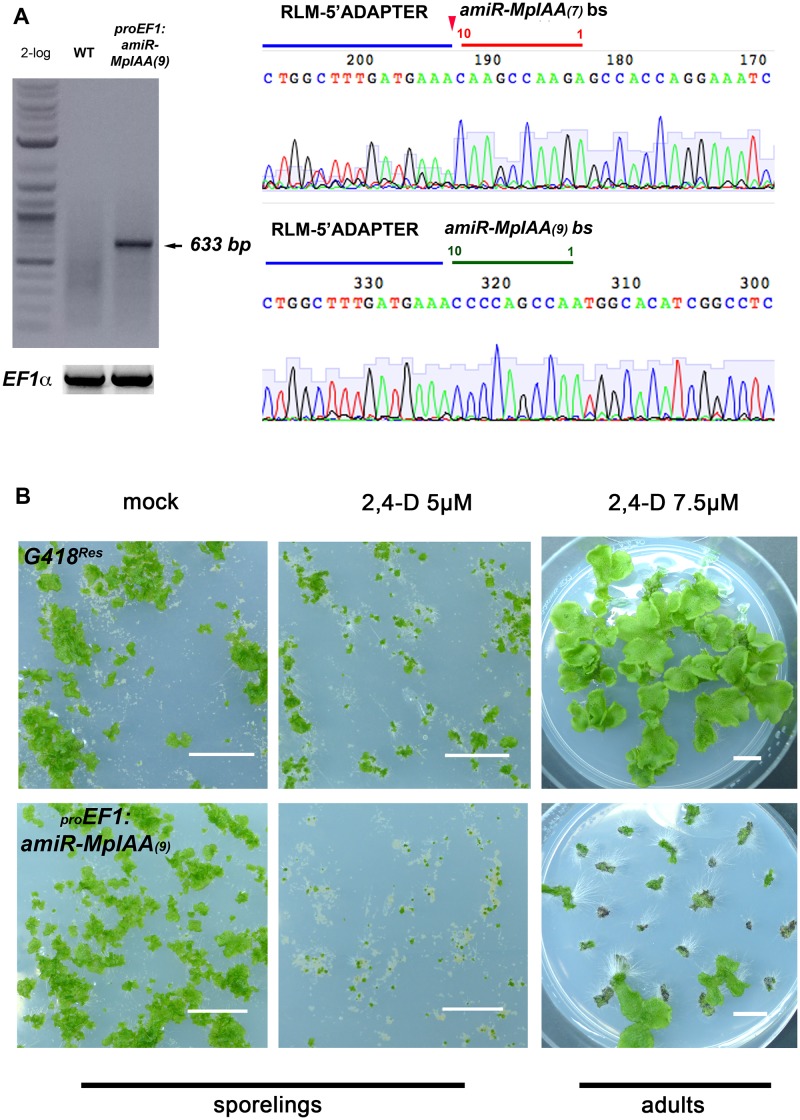
*MpIAA* represses auxin signaling in *M*. *polymorpha*. **(A)** RLM RACE of *amiRMpIAA* expressing lines. Left panel shows gel electrophoresis of RLM-RACE products. Right panel shows direct sequencing of the 633bp RLM-RACE product from *amiRMpIAA*_*9*_. Sequencing detected the expected cleavage of *MpIAA* between bases 10/11 of both miR sequences. **(B)** Auxin hypersensitivity in knock-down *MpIAA* lines at both the sporeling and adult stages. At the sporeling stage, _*pro*_ *EF1*:*amiRMpIAA*_*9*_ sporelings grown on 5μM 2,4-D from germination have severely affected area compared to controls. In the adult stage plants were grown for 16 days on B5 media prior to treatment with 2,4-D. _*pro*_ *EF1*:*amiRMpIAA*_*9*_ treated with 7.5μM 2,4-D for ten days form ectopic rhizoids compared to controls. In all panels, each of the plants is an independent transformant. Scale Bars = 1 cm.

To test whether the components identified by homology act in an auxin signaling pathway, we created loss- and gain-of-function alleles of the genes. Two independent artificial microRNAs (amiRs) were designed to target *MpIAA (amiRMpIAA*_*7*_ and *amiRMpIAA*_*9*_). For amiR design in *M*. *polymorpha*, we used the backbone of the endogenous *MpMIR160* precursor (S7 Fig), replacing the miR and miR* sequences with sequences to target the gene of interest, and cleavage was corroborated using RLM-RACE (Fig 2A; [68]). Both amiRs were constitutively expressed under the *EF1-*α promoter (*proEF1*) and the reduction of target MpIAA transcripts was corroborated (S8 Fig). While most _*pro*_ *EF1*:*amiRMpIAA*_*7*_ and _*pro*_ *EF1*:*amiRMpIAA*_*9*_ lines had nearly wild-type phenotypes, some lines exhibited a curly and convoluted thallus with long rhizoids, and *miRMpIAA* transcript levels are largely, albeit not perfectly, correlated with the severity of the phenotype (S8 Fig). Consistent with *MpIAA* having a role in auxin signaling, a majority of *amiRMpIAA*_*7/9*_ lines showed severe growth defects in exogenous auxin compared to wild-type controls (Fig 2). When grown in 2.5 and 5μM 2,4-D auxin media from the sporeling stage, _*pro*_ *EF1*:*amiRMpIAA*_*7/9*_ plants were dramatically smaller compared to wild-type controls, suggesting that exogenous auxin causes lethality when transcript levels of *MpIAA* are reduced (Fig 2B and S8D Fig). When mature _*pro*_ *EF1*:*amiRMpIAA*_*9*_ thalli were grown in mock media for 15 days and then transferred to 7.5μM 2,4-D plates for 20 days, loss of *MpIAA* resulted in profuse rhizoid production and a decrease in growth compared to controls (Fig 2B). In summary, knock-down *MpIAA* lines were auxin hypersensitive, indicating that *MpIAA* represses the auxin signaling pathway in *M*. *polymorpha*.

### MpARF1 is a modulator of the auxin signaling pathway

**Fig 3:**
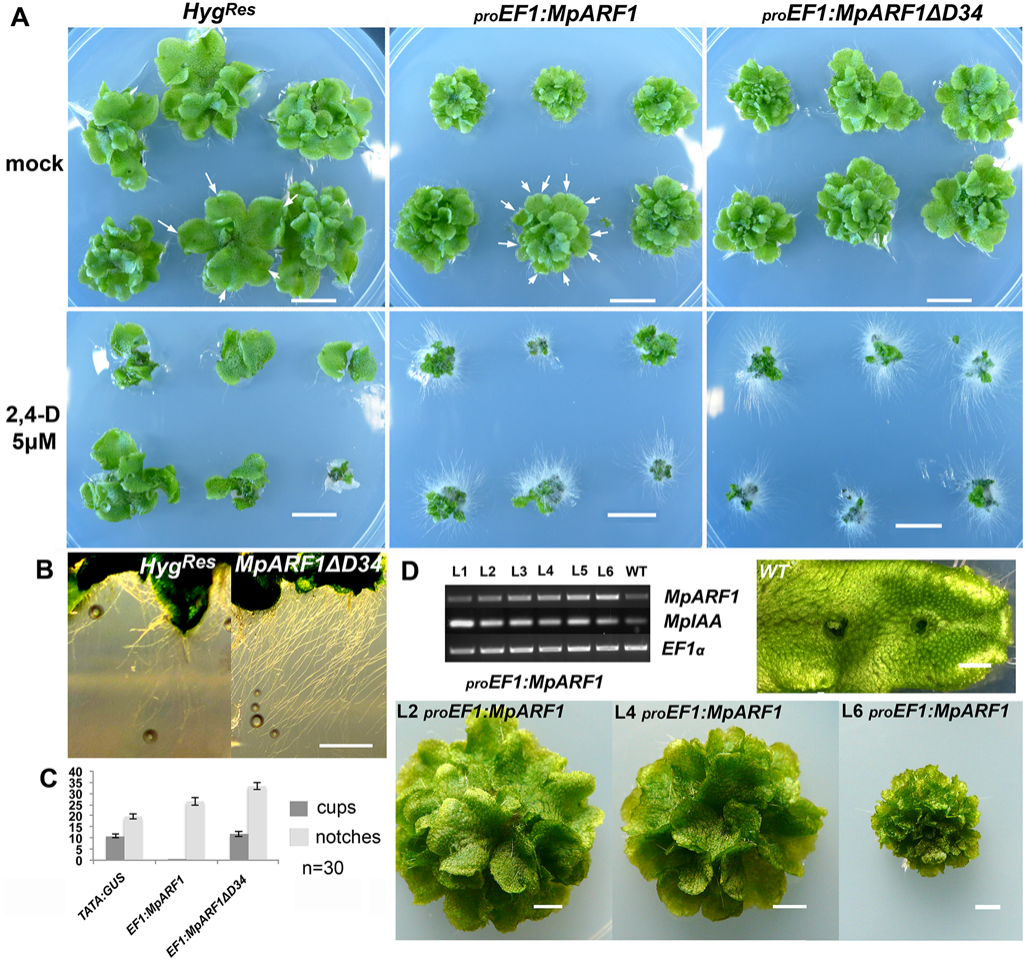
Gain-of-function *MpARF1* lines are auxin hypersensitive. **(A)** Each panel show six independent lines, transformed with constructs as indicated above the panels. Plants are treated as indicated to the left. Arrows indicate apical notches. **(B)** Left panel: Rhizoids from control Hyg^R^ lines. Right panel: _*pro*_ *EF1*:*MpARF1* ^*ΔD34*^ lines develop elongated and profuse rhizoids. **(C)** Apical notch and gemmae cup measurements for control, _*pro*_ *EF1*:*MpARF1* and _*pro*_ *EF1*:*MpARF1* ^*ΔD34*^ primary transformants after 50 days of growth (30 independent lines were scored per genotype). *MpARF1* overexpression results in increased branching rates. Deletion of D34^MpARF1^ recovers the gemmae cup production lost by over-expressing full length *MpARF1* transcripts. **(D)** Semi-quantitative RT-PCR showing six independent _*pro*_ *EF1*:*MpARF1* lines exhibiting an inverse correlation between *MpARF1* and *MpIAA* transcript levels. Three of the six lines are shown in the panel, with line L6 having more extensive branching and a smaller size compared to line L2. The constitutive translation *ELONGATION FACTOR 1-alpha (EF1)* was used as a control. All scale bars = 1 cm, except D = 1 mm.

To elucidate whether auxin hypersensitivity observed in *MpIAA* knockdown lines was due to misregulation of *ARF* genes, we explored the functional role of *MpARF1*. We focused on *MpARF1* since *MpARF1* transcripts are readily detectable in the thallus, whereas we did not detect transcripts of *MpARF2* in the thallus (S8B Fig). Evidence that MpARF1 elicits a transcriptional output of the auxin signaling pathway comes from mutant alleles obtained by CRISPR-CAS (Sugano et al 2014) and artificial miRNAs [69], where plants deficient in *MpARF1* activity are unable to form ectopic rhizoids in the presence of exogenous auxin. Our phylogenetic analysis places *MpARF1* as a member of class A *ARF* activators, loss-of-function alleles of which cause auxin insensitivity in *Arabidopsis*. Thus, gain-of-function *MpARF1* alleles should recapitulate aspects of loss-of-function *MpIAA* alleles. We cloned a wild-type allele of *MpARF1* as well as a truncated dominant gain-of-function mutant of *MpARF1* lacking D34 (*MpARF1* ^*ΔD34*^; S9 Fig) that should produce a protein with limited interaction with MpIAA (Krogan et al 2012). When *MpARF1* and *MpARF1* ^*ΔD34*^ genes were driven by _*pro*_ *EF1-*α, plants displayed auxin hypersensitivity, exemplified by formation of ectopic rhizoids in adults exposed to 5μM 2,4-D (Fig 3A). Even in the absence of auxin, *proEF1*:*MpARF1* ^*ΔD34*^ plants exhibited excessive rhizoid production with rhizoids growing to greater lengths compared to a Hyg^Res^ control, visible when plants are grown on vertically oriented plates such that gravity influenced rhizoid growth is evident (Fig 3B). *proEF1*:*MpARF1* and *proEF1*:*MpARF1* ^*ΔD34*^ plants exhibited higher rates of branching, seen as a significant increase in apical notches compared to controls (Fig 3A and 3C; S3 Table), and *proEF1*:*MpARF1* ^*ΔD34*^ lines had increased gemma cup production compared to *proEF1*:*MpARF1* lines (Fig 3C). The extent of phenotypic aberration was correlated with *MpARF1* transgene expression (Fig 3D). Additionally, the levels of *MpIAA* expression appear to be inversely correlated with levels of *MpARF1* expression, supporting the idea that *MpARF1* inhibits transcription of *MpIAA*

### MpTPL is involved in patterning multiple tissues in *M*. *polymorpha* development

**Fig 4:**
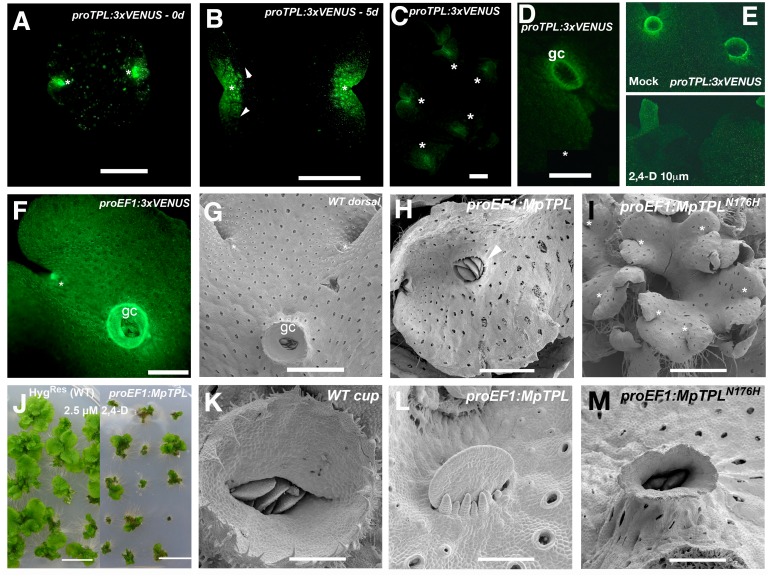
Developmental role of *MpTPL* in *M*. *polymorpha*. **(A)** Mature dormant gemma of _*pro*_ *MpTPL*:*3xVENUSN7* with expression in two apical notches (asterisks). **(B)** 5-day-old gemmae of _*pro*_ *MpTPL*:*3xVENUSN7*. Expression has expanded from apical notches (asterisk) to zones of newly formed air chambers (arrowheads). Inset illustrates background fluorescence in the wild type with identical settings. **(C)** Mature 25-day-old _*pro*_ *MpTPL*:*3xVENUSN7* line showing expression in meristematic regions (asterisks). **(D)** Young gemmae cup (gc) primordia in mature _*pro*_ *MpTPL*:*3xVENUSN7* line. **(E)** Expression pattern of mature _*pro*_ *MpTPL*:*3xVENUSN7* thallus on mock and 10μM 2,4-D media. Plants were transferred to exogenous auxin after 16 days on B5 media. **(F)**_*pro*_ *EF1*:*VENUS* indicating expression in apical notches (asterisk) and gemmae cups (gc). **(G)** Dorsal side of wild-type thallus covered with photosynthetic air chambers and two apical notches from which gemmae cups (gc) develop. **(H)**_*pro*_ *EF1*:*MpTPL* plants show dorsiventrality but are affected in cup development (arrowhead). **(I)** Representative _*pro*_ *EF1*:*MpTPL* ^*N176H*^ line with a compact and multi-bifurcated thalli, with at least 7 apical notches (asterisks) produced in a similar area compared to (G). **(J)**_*pro*_ *EF1*:*MpTPL* plants have diminished area in low auxin concentrations compared to controls. Each plant is an independent primary transformant grown for a month on 2.5μM 2,4-D. **(K)** Wild-type mature gemmae cup harboring dormant gemmae. **(L)**_*pro*_ *EF1*:*MpTPL* plants have no gemmae cups, instead naked gemmae and gemmae primordia with filaments growing by transversal cell divisions can be seen. **(M)**_*pro*_ *EF1*:*MpTPL* ^*N176H*^ gemmae cups are elongated and narrow, similar to developmental effects produced by exogenous auxin application in the wild type. Scale bars in A and B = 0.5mm; C, D, E, F, H, M = 1mm; G and I = 1.5mm; J = 1 cm; K = 300μm; L = 75μm; M = 0.6mm.

The lack of strong phenotypic effects in knock-down *MpIAA* lines prompted us to examine the single member of the *TOPLESS* corepressor family in *M*. *polymorpha*. Expression patterns of a _*pro*_ *MpTPL*:*3xVENUS* fusion, including 3.6kb upstream of *MpTPL* transcript up to the nearest upstream transcript, exhibited intense fluorescent signal in the meristematic regions during most developmental stages, including mature gemmae (Fig 4A), five-day-old gemmalings (Fig 4B), and mature 25-day-old thalli (Fig 4C) compared to autofluroesence in the wild type (S10A Fig). Expression was also detected in gemmae cups (Fig 4D). Transfer of mature _*pro*_ *MpTPL*:*3xVENUS* plants to media containing 10 μM 2,4-D resulted in a decreased level of expression of the reporter gene, suggesting regulatory feedback between auxin and *MpTPL* (Fig 4E). Expression of _*pro*_ *MpTPL*:*3xVENUS* was observed throughout the thallus, although the signal was weaker compared to _*pro*_ *EF1*:*3xVENUS* lines (Fig 4F).

The wild-type dorsiventral *M*. *polymorpha* thallus is several cell layers thick. The single apical cell in the shoot mersitem has four cleavage planes: one dorsal, one ventral, and two lateral [70,71]. Dorsal and lateral derivatives contribute to the formation of dorsal photosynthetic air chambers and gemmae cups (Fig 4G). Notably both dorsal gemmae cups and ventral rhizoids are produced along a midline (Fig 4F and 4G, and S2 Fig). Wild-type gemmae cups produce a characteristic cup rim that is several cell layers thick at its base and unistratose towards its apex and acts to store and protect dormant gemma (Fig 4K).

Ectopic expression of wild-type *MpTPL* using *proEF1-*α resulted in slightly smaller and darker green thalli able to form a bifurcating thallus with normal dorsiventral polarity (Fig 4H and S10B Fig). However, gemmae cup formation was disrupted in 80% of _*pro*_ *EF1*:*MpTPL* lines (N = 15), with gemmae cups entirely absent in some cases (Fig 4H and 4L). The loss of gemmae cups did not impair the formation of gemmae, as naked gemma and gemma primordia developed in the positions where gemmae cups would normally develop along the midline (Fig 4K and 4L), reminiscent of pharmacological effects of L-Kyn on the thallus. Intriguingly, _*pro*_ *EF1*:*MpTPL* lines were hypersensitive to 2.5 μM exogenous 2,4-D, similar to knock-down *MpIAA* lines (Fig 4J).

We created a putative *MpTPL* loss-of-function allele analogous to the dominant negative *Arabidopsis TPL* ^*(N176H)*^ and drove its expression with _*pro*_ *EF1* [9]. The most conspicuous phenotype of _*pro*_ *EF1*:*MpTPL* ^*N176H*^ plants was an increased frequency of thallus branching compared to wild type and _*pro*_ *EF1*:*MpTPL* plants (Fig 4I; S2 Table). In this regard _*pro*_ *EF1*:*MpTPL* ^*N176H*^ plants resemble *MpARF1* overexpression lines (Fig 4M). Thalli of _*pro*_ *EF1*:*MpTPL* ^*N176H*^ plants produced nearly three apical notches in the time in which wild-type plants produce one apical notch (Fig 4G and 4I). _*pro*_ *EF1*:*MpTPL* ^*N176H*^ plants had diminished production of gemmae cups, and when produced, gemmae cups were aberrant, being narrow and elongate, reminiscent of gemmae cups that develop in the presence of exogenous auxin (Fig 4M). When reproductive development was induced under far-red light _*pro*_ *EF1*:*MpTPL* ^*N176H*^ gametophore stalks showed decreased elongation compared to wild type, resulting in gametophores of short stature and with dorsal protrusions reminiscent of rhizoids (S10C Fig).

### Auxin insensitive *M*. *polymorpha* plants fail to establish a dorsiventral thallus

In *Arabidopsis* translational fusions of TPL and D34 of AUX/IAA proteins, such as IAA12/BODENLOS (BDL; TPL-D34^BDL^), constitutively bind to ARF5/MONOPTEROS (MP) via interactions with D34, keeping MP in a repressive complex even in the presence of auxin [9]. Given the putative role of MpIAA and MpTPL proteins in auxin signaling in *M*. *polymorpha*, chimeric translational fusions of MpTPL to D34 of all *M*. *polymorpha* loci bearing putative D34 of protein-protein interaction were generated and all fusions were regulated by _*pro*_ *EF1*, making sure non of the fusions carried an endogenous miR binding site (S11 Fig; [40]).

**Fig 5:**
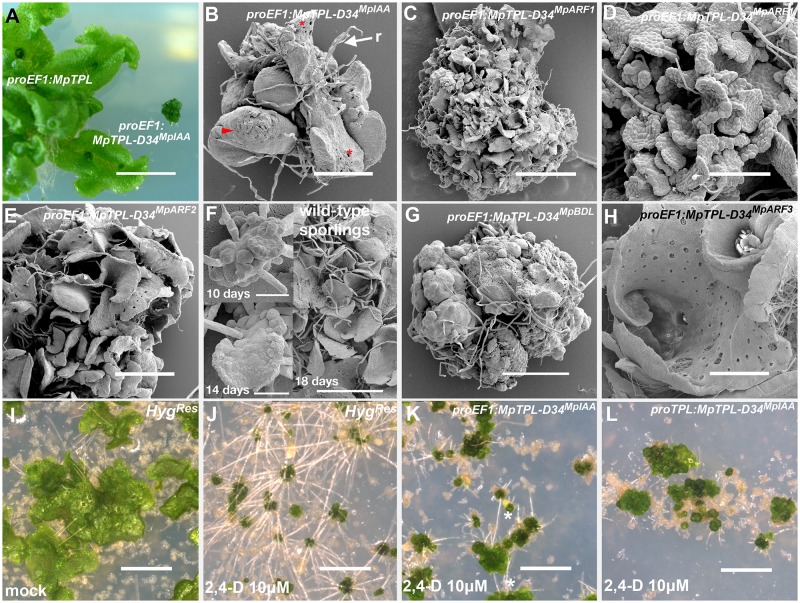
Developmental effects of auxin insensitivity. **(A)**_*pro*_ *EF1*:*MpTPL-D34* ^*IAA*^ lines suffer from severe dwarfism compared to _*pro*_ *EF1*:*MpTPL* plants. **(B)** Strong _*pro*_ *EF1*:*MpTPL-D34* ^*IAA*^ line showing a lack of organized patterning, dorsiventral polarity and a limited degree of differentiation. Scales (arrowhead), air pores (asterisk) and rhizoids (r) are visible in random places and growth is severely affected. **(C)**_*pro*_ *EF1*:*MpTPL-D34* ^*MpARF1*^ representative line showing a degree of differentiation similar to the prothallus stage of wild-type development. **(D)**_*pro*_ *EF1*:*MpTPL-D34* ^*MpARF1*^ showing single cell layer prothalli-like tisssue. **(E)**_*pro*_ *EF1*:*MpTPL-D34* ^*MpARF2*^ representative line. **(F)** Wild-type sporeling (10 days) transitioning into a single cell layered prothallus (14 days) and fully established multiple prothalli 18 days after plating. **(G)** Strong _*pro*_ *EF1*:*MpTPL-D34* ^*BDL*^ line lacking growth, differentiation and dorsiventrality, but still capable of producing rhizoids. **(H)**_*pro*_ *EF1*:*MpTPL-D34* ^*MpARF3*^ lines differentiate gemmae cups and a dorsiventral polarity with dorsal air chambers and ventral rhizoids. The thallus has an apparent hyponastic curvature. **(I, J)** Multiple independent Hyg^Res^ sporelings (transformed with an empty binary vector) grown on media containing 10μg/ml Hyg and mock (**I**) or 10μM 2,4-D (**J**) for 14 days. **(K, L)** Multiple independent _*pro*_ *EF1*:*MpTPL-D34* ^*MpIAA*^ (**K**) or _*pro*_ *MpTPL*:*MpTPL-D34* ^*MpIAA*^ (**L**) lines grown on 10μM 2,4-D for 14 days. Scale bars in C, E, I, J, K and L = 1mm; A = 1cm; B = 430μm; D = 130μm; F = 30μm; G = 600μm; H = 860μm.

_*pro*_ *EF1*:*MpTPL-D34* ^*MpIAA*^ plants exhibited severe dwarfism (Fig 5A) and failed to establish a dorsiventral thallus. Individuals were composed of a mass of undifferentiated cells and incompletely developed organs with random orientations (Fig 5B). Occasional rhizoids, scales and air chambers were observed, but these structures were not organized relative to one another as in wild-type plants (Fig 5B). Similar phenotypes were observed for _*pro*_ *EF1*:*MpTPL-D34* ^*MpARF1*^ (Fig 5C and 5D), _*pro*_ *EF1*:*MpTPL-D34* ^*MpARF2*^ (Fig 5E). A majority of these lines only achieved the level of differentiation of the sporeling or prothallus stages of wild-type development (Fig 5F), with individuals not transitioning further than single cell layer tissues (Fig 5D). Fusions of TPL with D34 of BDL (_*pro*_ *EF1*:*MpTPL-D34* ^*BDL*^) resulted in similar phenotypes, suggesting a high degree of functional conservation between liverwort and angiosperm D34^IAA^ protein domains (Fig 5G). Weaker _*pro*_ *EF1*:*MpTPL-D34* ^*BDL*^ lines had a narrow thallus with fused gemmae cups, as in L-Kyn treatments and in lines expressing the IAA conjugating enzyme iaaL (S12A and S12B Fig).

In contrast to the severe developmental defects described above, _*pro*_ *EF1*:*MpTPL-D34* ^*MpARF3*^ plants (Fig 5H) did not exhibit altered patterning in a manner comparable to other chimeric genes tested. _*pro*_ *EF1*:*MpTPL-D34* ^*MpARF3*^ thalli had a convoluted dorsal surface (Fig 5H and S12C Fig), with thallus margins curled inwardly towards the midline, especially close to the apical notches, resulting in a more compact thallus than that of wild type (Fig 5H). _*pro*_ *EF1*:*MpTPL-D34* ^*MpARF3*^ thalli produced ventral rhizoids and dorsal air chambers and gemmae cups. However, _*pro*_ *EF1*:*MpTPL-D34* ^*MpARF3*^ plants grew bigger than other *TPL-D34* fusions (S12C Fig).

As MpTPL acts as a repressor of auxin transcriptional response, we drove expression of the chimeric proteins with the *MpTPL* regulatory sequences. _*pro*_ *MpTPL*:*MpTPL-D34* ^*MpIAA*^ lines had a similar, but slightly milder, phenotype than _*pro*_ *EF1*:*MpTPL-D34* ^*MpIAA*^ lines: plants failed to pattern a flattened dorsiventral thallus and also formed a cluster of organs similar to wild-type prothalli (Fig 5F). Some _*pro*_ *MpTPL*:*MpTPL-D34* ^*MpIAA*^ lines however were able to form a single gemma cup able to produce gemmae (S12D and S12E Fig). The gemmae from these cups had distorted shapes with marginal extensions, and lacked the two apical notches conspicuous on wild-type gemmae (S12E Fig).

One prediction of constitutively expressing MpTPL-D34 chimeric proteins is a downregulation of activator ARF activity even if MpIAA proteins are degraded due to the presence of auxin, i.e. the transgenic plants should be auxin insensitive. When grown in the presence of exogenous auxin _*pro*_ *EF1*:*MpTPL-D34* ^*MpIAA*^ plants occasionally produce ectopic rhizoids compared to controls (Fig 5I–5K), while expression of *MpTPL-D34* ^*MpIAA*^ using _*pro*_ *MpTPL* produced auxin insensitive plants as assayed by the lack of ectopic rhizoid formation (Fig 5L). These results indicate that expression of the chimeric MpTPL-D34^MpIAA^ protein is capable of creating auxin insensitive plants if it expressed in the appropriate cells at appropriate levels.

## Discussion

### A minimal auxin signaling network is sufficient to pattern a complex body plan

Phylogenetic analyses indicate the *M*. *polymorpha* genome possesses orthologues encoding components of the auxin transcriptional response machinery as it has been characterized in other land plants [72,73,74]. In contrast to other land plant lineages only single orthologs of each of the three classes of *ARF* and single orthologs of the *AUX/IAA*, *TIR1/AFB*, and *TPL* gene families are present in *M*. *polymorpha*. Phylogenetic analyses also suggest that the last common ancestor of land plants had single orthologs of these classes of genes, implying that *M*. *polymorpha* has retained the ancestral condition. Single orthologs of other regulatory gene families, e.g. the class III and IV HD-ZIP transcription factor families, in the last common ancestor of land plants have also been proposed and may be a common feature of the ancestral land plant [75,76]. As the genome of the filamentous charophycean alga *Klebsormidium* does not encode any of the canonical auxin transcriptional response machinery [77], its origin must lie either at the base of the land plant lineage or along the charophycean algal grade leading to land plants, as is the case for several other transcriptional regulators [78].

In flowering plants, the complexity and the robustness of the auxin transcriptional response has been hypothesized to be generated by a large number of signaling network paralogs encoding components of differing affinities [10,24]. However, the relative transcriptional simplicity of the auxin response system in *M*. *polymorpha* is sufficient to pattern its complex thallus with several tissue and cell types. While the common ancestor of mosses and flowering plants is also predicted to have possessed only single orthologs of the genes in question, relatively recent gene duplications in the lineage leading to the moss *P*. *patens* has resulted in this species possessing multiple closely related paralogs [74]. As both *M*. *polymorpha* and *P*. *patens* have arguably similar levels of complexity at the gametophytic level, our findings support the idea that the relationships between auxin signaling components—rather than the number of paralogs—are the key to establish a complex body plan. Regarding the sporophyte generation, it is still possible to speculate that increases in the numbers of paralogs of the auxin genetic toolkit could be correlated with increased morphological complexity but this requires further sampling of land plant genomes, in particular those of hornworts.

### Conserved auxin transcriptional machinery in *M*. *polymorpha*

Several lines of evidence indicate a conserved role for *MpIAA*, *MpARF1* and *MpTPL* in auxin response. First, these genes are orthologous to those that have been shown to act downstream of auxin in developmental and physiological processes in other land plants (S4–S6 Figs). Second, similar phenotypic defects are seen in *M*. *polymorpha* when expressing MpTPL-D34 translational fusions with D34 derived from either the *Arabidopsis* AUX/IAA protein BDL, or the *M*. *polymorpha* MpIAA, MpARF1 or MpARF2 proteins (Fig 3D–3F). Third, _*pro*_ *MpTPL*:*MpTPL-D34* ^*IAA*^ *M*. *polymorpha* plants are insensitive to exogenous auxin, similar to the corresponding plants in *Arabidopsis* [9]. Fourth, *MpTPL-D34* alleles and loss- and gain-of-function alleles of *MpIAA*, *MpARF1* and *MpTPL* recapitulate phenotypes observed when *M*. *polymorpha* is grown in the presence of either exogenous auxin or auxin biosynthesis inhibitors (Fig 1), as is discussed in detail below. Fifth, reduction of *MpIAA* transcripts or ectopic expression of *MpARF1* and *MpTPL* results in auxin hypersensistivity (Figs 2, 3 and 4J). Sixth, loss-of-function alleles of *MpARF1* result in auxin hyposensitivy [69,79]. Taken together, these data strongly suggest that the *MpTPL*, *MpARF1*, and *MpIAA* genes encode components of an auxin transcriptional response system in *M*. *polymorpha*.

### The auxin transcriptional response machinery in land plants

Our data in *M*. *polymorpha* support aspects of the model proposed for *Arabidopsis* explained in detail in the introduction. That indistinguishable phenotypes were observed when any of the chimeric proteins *MpTPL-D34* ^*MpIAA*^, *MpTPL-D34* ^*MpARF1*^, or *MpTPL-D34* ^*MpARF2*^ were constitutively expressed suggests MpARF1 can homodimerize as well as heterodimerize with MpIAA and MpARF2 *in planta*. Y2H and in BiFC experiments on these proteins also suggest that they can all interact with one another, with the strongest interactions occurring between MpIAA and MpARF1 [67]. Furthermore, that _*pro*_ *TPL*:*MpTPL-D34* ^*MpIAA*^ plants are insensitive to auxin indicates that interactions between MpIAA, MpARF1, and MpARF2 mediate the activation of gene expression in response to auxin in *M*. *polymorpha*. In contrast, constitutive expression of MpTPL-D34^MpARF3^ chimeric proteins does not inhibit establishment of dorsiventrality or differentiation of tissues, suggesting MpARF3 does not interact effectively with MpARF1 and MpIAA proteins, pointing to a possible role of class C ARFs regulating gene expression in an auxin independent manner. Consistent with this hypothesis, is the observation that MpARF3 can’t homodimerize and its interaction with MpIAA is weaker than the interactions between MpIAA and either MpARF1 or MpARF2 [67].

### Auxin patterns the development of many tissues in *M*. *polymorpha*

A large body of literature, primarily of pharmacological experiments whereby *M*. *polymorpha* has been grown in the presence of exongenous auxins, anti-auxins, and auxin transport inhibitors, has indicated auxin is involved in rhizoid formation, establishment of dorsiventral polarity in gemmalings, polar thallus regeneration, gemma and apical cell dormancy, gemmae cup development, air pore development, and spacing of organs during branching [40,41,42,43,44,45,46,47,48,49,50,51,52,53,54,80]. As outlined below, our pharmacological and genetic studies confirm and extend the known roles of auxin in the development of multiple structures during several stages of *M*. *polymorpha* development. For the sake of clarity, the different experiments and their putative effects performed in this study are summarized in S4 Table.

#### Dorsiventral polarity

Auxin distribution influences establishment of the dorsiventral body plan. Consistent with earlier reports, growth of gemmae from germination on auxin containing media resulted in rhizoid formation from both dorsal and ventral surfaces. In addition, transfer of gemmae with established dorsiventrality to auxin plates caused several other developmental abnormalities, such as distorted growth of air chambers, air pores, and gemmae cups, but not changes in dorsiventrality (Fig 1A–1H). This is consistent with the observation that dorsiventrality is established within the first 24 hours of gemmaling growth and is irreversible after this time [32,45,81,82,83,84,85].

#### Rhizoids

Rhizoid formation is promoted by auxin in receptive tissues. When gemmae are germinated in the presence of exogenous auxin, cells on both the dorsal and ventral surfaces have the potential to differentiate into rhizoids, however, once dorsiventrality is established, dorsal cells lose the capacity to form rhizoids in response to high levels of auxin. However, in gemmalings with established polarity, auxin induces production of additional central and ventral rhizoids and they grow to greater lengths (S1B Fig). Conversely, when gemmae are grown in the presence of auxin biosynthesis inhibitors, fewer, and sometimes shorter, rhizoids develop from the central surface (Fig 1K and 1L). Finally, _*pro*_ *MpTPL*:*TPL-D34* ^*IAA*^ plants do not form rhizoids even in the presence of exogenous auxin.

#### Gemmae and gemmae cups

Gemmae cups are one of the most sensitive tissues to disruptions in auxin homeostasis. When grown in the presence of auxin biosynthesis inhibitors gemmae cup and gemmae formation is delayed (Fig 1I and 1J). Over-expression of *MpTPL* (as in _*pro*_ *EF1*:*MpTPL* lines), which presumably results in a dampening of auxin transcriptional response, also results in a reduction in gemmae cup growth (Fig 4H, 4K and 4L). In contrast, when thalli with established polarity are treated with exogenous auxin elongated tubular cups are formed (Fig 1E–1H). The tubular gemmae cups often show a gravitropic response opposite to the usual negative gravitropism previously described for gemma cup growth [55], and consistent with the notion that auxin transduces a gravity stimulus or inhibits a phototropic response [86,87]. Excess auxin may be transported into gemmae cups, or alternatively, auxin-conjugating enzymes (such as *GH3*) may metabolize auxin in most other tissues except the gemmae cup walls.

#### Branching

Auxin influences the rate of branching during thallus growth. Growth of the *M*. *polymorpha* thallus occurs via divisions of a single apical cell with four cutting faces [70,71]. Branching is dichotomous (S2 Fig), but the precise mechanism, e.g. via division of the apical cell into two apical cells or *de novo* formation of nearby apical cells, is not known. Application of the auxin biosynthesis inhibitor L-Kyn and expression of a bacterial auxin-conjugating enzyme (iaaL) appear to slow the rate of branching. L-Kyn in particular, causes a disruption of the growth phase that spatially separates apical notches (Fig 1M, 1N and 1R). Thalli often have gemmae cups produced by two adjacent apices fused into a single structure, suggesting fields of cells can act coordinately in the development of a single gemmae cup, indicating *M*. *polymorpha* exhibits a remarkable plasticity during organogenesis. In contrast, an increase in the rate of branching results from over-expression of *MpARF1* and *MpARF1* ^*ΔD34*^ (Fig 3A and 3C; S3 Table) as well as the putative reduction in *MpTPL* activity caused by the dominant negative construct _*pro*_ *EF1*:*MpTPL* ^*N176H*^ (Fig 4I; S2 Table). Because the overexpression of an MpIAA-insensitive MpARF1^ΔD34^ protein results in an enhanced phenotype compared to the one observed in *MpARF1* overexpressing lines, it is unlikely that these lines have secondary loss-of-function effects as a result of increased *MpIAA* transcription [11,12,20,21,88]. Thus, MpARF1 facilitates activities as diverse as cell expansion (expansion of rhizoids) and pluoripotency reprograming (bifurcation).

#### Developmental stage transitions

Auxin is required for gametophyte developmental stage transitions. Several different *MpTPL-D34* lines, especially in the cases of _*pro*_ *EF1*:*MpTPL-D34* ^*MpARF1*^, and _*pro*_ *EF1*:*MpTPL-D34* ^*MpARF2*^, phenotypically resemble wild type at the prothallus stage (Fig 5B–5F), with a multitude of undifferentiated unistratose lobes composed (Fig 5D). Additionally, the phenotypes of strong _*pro*_ *EF1*:*iaaL* lines are reminiscent of wild-type sporelings, prior to transitioning into a prothallus (Figs 1O, 1P and 5F). These results suggest that auxin is required for the morphological transitions between successive developmental stages during *M*. *polymorpha* gametophyte development—from sporeling to prothallus, which can be viewed as a transition from unorganized growth to 2-dimensional patterning, and from prothallus to thallus, which is a transition from 2-dimensional to 3-dimensional patterning. The requirement of auxin for developmental transitions has been observed in another bryophyte lineage, the mosses, where auxin is necessary to produce 3-dimensional gametophores from protonemal filaments [25,64]. The ubiquitous requirement for auxin for morphological transitions during development of these basal lineages of land plants suggests that the common ancestor of embryophytes likely utilized auxin as an internal cue to promote morphological transitions. Given the origin of the auxin transcription response machinery was at or near the origin of land plants and that a key innovation of land plants is development of 3-dimensional bodies from an apical cell of at least three cutting faces, it is plausible that the incorporation of auxin response into developmental programs facilitated the evolution of complex land plant body plans.

#### Organized tissue patterning

In many _*pro*_ *EF1*:*iaaL* lines there is little, if any, organized tissue growth even though the plants continue to grow for months, suggesting that a reduction in auxin levels, that while not inimical to life, results in an almost complete loss of tissue patterning in *M*. *polymorpha* (Fig 1). Auxin insensitive _*pro*_ *MpTPL*:*MpTPL-D34* ^*IAA*^ and _*pro*_ *EF1*:*MpTPL-D34* ^*IAA*^ plants can produce many of the tissues found in a wild-type thallus, including air chambers, air pores, gemmae cups and gemmae. However, the organization of the tissues relative to one another and of the tissues themselves is severely disrupted (Fig 5B–5G). Such distortions of tissue patterning, (e.g. shapes of air chambers, air pores, gemmae cups), are also observed when thalli are grown in the presence of exogenous auxin or auxin biosynthesis inhibitors. Taken together, these data suggest that auxin, or processes downstream of auxin are required for not only developmental transitions, but also for the patterning of tissues relative to each other and development most tissue types in the *M*. *polymorpha* gametophyte.

In a classic experiment nearly a century ago, geotropic sensitivity to decapitated roots was restored by placing either another root tip or, alternatively, a shoot tip on the decapitated stump, thus demonstrating that auxins produced in the shoot and root tips do not have specificity of action, but rather act in a context dependent manner [89]. Given that auxin acts in the patterning of most tissue and cell types of the *M*. *polymorpha* gametophyte and the lack of redundancy in its components of the auxin transcriptional response machinery, there appears to be no tissue or cell type specificity of action with respect to auxin signaling components. Rather, it seems that since the origin of land plants, auxin acted as a facilitator rather than a determiner of cell fate, with the specific output of ARF action depending upon the milieu of other transcription factors present in a particular cell [90,91].

## Materials and Methods

### Plant growth conditions

Sporangia from an Australian population of *Marchantia polymorpha* (from now on referred to as wild type) were collected from a southeastern Melbourne field location (37°57'48.36"S, 145° 6'20.41"E). Sporangia were sterilized in 1x sterilization solution (2% sodium hypochlorite, 0.1% TritonX-100) for 1 minute and subsequently washed with water. Sporangia were then dried and stored at 4°C.

Wild-type spores were suspended in water and plated on petri dishes with Gamborg’s B5 basal medium (B5; Austratech,). Dormant gemmae from adult plants were subcultured for pharmacological assays. Spores were also grown in liquid 0M51C media [92] for transformation (see transformation section below).

### Pharmacological assays

Dormant gemmae, or gemmalings grown on B5 media for 2, 4, 6, 8, 10, 12, 14 and 16 days, were plated on B5 media with 10 μM 2,4-D or NAA (Sigma-Aldrich). L-Kyneurenine (Sigma-Aldrich) was used in a similar fashion as exogenous auxin.

### Phylogenetic analyses

Nucleotide sequence alignments for seven species with complete genomes (*Physcomitrella patens*, *Selaginella moellendorffii*, *Picea abies*, *Pseudotsuga menziesii* (used only for *TPL)*, *Amborella trichopoda*, *Solanum lycopersicum* and *Arabidopsis thaliana)* plus the isolated *M*. *polymorpha* genes (see below) were created manually using Se-Alv2.0a11 [93]. Ambiguous alignment regions were trimmed for phylogenetic inferences. Bayesian phylogenies were performed using Mr. Bayes 3.2.1 [94]. A nucleotide *TPL* phylogeny with 3111 characters and 23 taxa was performed assuming variable rates between first, second and third positions (prset ratepr = variable). The analysis was run for 500,000 generations, sufficient for two simultaneous runs to converge with statistical significance (P <0.01). A nucleotide AUX/IAA phylogeny with 309 characters and 49 taxa was performed assuming variable rates between first, second and third positions (prset ratepr = variable). The analysis was run for 5,000,000 generations, sufficient for two simultaneous runs to converge with statistical significance (P <0.01). For the ARF phylogeny, a nucleotide matrix with embryophyte sequences (44 taxa, 996 characters) were run in Mr. Bayes for 750,000 generations assuming variable rates between first, second and third positions (prset ratepr = variable). Each analysis was run until the standard deviation of split frequencies dropped below 0.01. For all phylogenies, 25% of trees were discarded in the burn-in phase.

### Cloning and plasmids

All primer sequences are available in S5 Table. The *MpEF1* promoter was amplified with primers MpEF1-NdeI-F and MpEF1-SalI-R, cloned into pCRII-TOPO (Invitrogen) and sequenced. _*pro*_ *EF1* was subcloned into the shuttle vector BJ36 using *Nde*I and *Sal*I. The resulting plasmid was called _pro_EF1 BJ36 and was used to create constitutive expression constructs described below.

#### Cloning of *MpSHI* promoter and _*pro*_ *MpSHI*:*GUS*

A 3981bp promoter/UTR fragment of the single *SHI/STY* ortholog in *M*. *polymorpha*, *MpSHI*, was amplified using primers ME1 and ME2. The fragment was cloned in pCRII-TOPO, creating pME68, and subsequently transferred to pRITA, which has MCS upstream of a GUS reporter gene and transcriptonal terminator sequence [95] using *Nde*I and *Kpn*I, creating pME82. A _*pro*_ *MpSHI*:*GUS Not*I fragment from pME82 was later subcloned to pSKF HART. pSKF HART, created by Dr. Sandra Floyd, is a binary vector based on pBART (used for *Arabidopsis* transformation), but in contrast to the BASTA/Glufosinate resistance gene in pBART, HART has two CaMV35S promoters in tandem driving an *hptII gene* (Hygromicin resistance). An NdeI/KpnI _*pro*_ *MpSHI* fragment from pME68 was subcloned into _pro_EF1 BJ36, replacing the *EF1* promoter, resulting in plasmid pME159.

#### *EF1*:*iaaL* and *MpSHI*:*iaaL* constructs

A bacterial iaaL CDS was amplified from plasmid iaaL-BJ36 with primers ME83 and ME84. The resulting fragment was cloned in pCRII-TOPO and sequenced. *iaaL* was subcloned into _pro_EF1 BJ36 and pME159 using *Kpn*I and *Hin*dIII. The resulting _*pro*_ *EF1*:*iaaL* and _*pro*_ *MpSHI*:*iaaL* fragments were then subcloned into pSKF HART.

#### Cloning and mutagenesis of *MpTPL*

A 1kb *MpTPL* fragment was cloned from wild-type *M*. *polymorpha* thallus cDNA using a nested PCR approach with degenerate primers TPL-F1, TPL-F2, TPL-R1 and TPL-R2, flanking a conserved region in TPL transcripts. The obtained fragment was cloned in pCRII-TOPO and sequenced. For 5’RLM-RACE outer primers (UPM and TPLR4) and inner primers (NUP and TPLR3) were used. For 3’RACE TPLF3, TPLF4 and a GAGA primer were used. All fragments were cloned in pCRII-TOPO and sequenced. A full-length transcript was amplified using primers MpTPL-SalI-F and MpTPL-EagI-R and cloned and sequenced in pCRII-TOPO. This clone was named 1416G. PCR based mutagenesis was used to create *MpTPL* ^*N176H*^. An initial set of two PCR fragments were amplified using primers M13F and MpTPL(N176H)-R (fragment 1), and primers MpTPL(N176H)-F and M13R (fragment 2), using 1416G as template. Both fragments 1 and 2 were used as template in a subsequent PCR reaction and amplified with primers M13F and M13R. The PCR product (named 1626) was cloned in pCRII-TOPO and sequenced.

#### MpTPL functional constructs

*MpTPL* was cut from 1416G with *Eco*RI and inserted in EF1proBJ36V2.0 resulting in _pro_EF1:MpTPL BJ36. _*pro*_ *EF1*:*MpTPL* was subcloned into pSKF HART using *Not*I. *MpTPL* ^*N176H*^ was cloned into entry Gateway vector pE2B using *Eco*RI (replacing the *ccdB* gene), and subsequently recombined into PKIGWB2 (Gateway binary vector provided by Dr. Kimizune Ishizaki and Prof. Takayuki Kohchi).

To construct *MpTPL-D34* fusions, an *MpTPL* transcript without stop codon was amplified using primers MluI-MpTPL-ATG-F and EcoRI-MpTPL-NoSTOP-R and cloned into EF1-BJ36V2.0 using *Mlu*I and *Eco*RI. D34 of *MpIAA* (*Eco*RI/*Hin*dIII), *MpARF1* (*Eco*RI/*Eco*RI), *MpARF2* (*Eco*RI/*Hin*dIII), *MpARF3* (*Eco*RI/*Hin*dIII) were amplified from wild-type thallus cDNA with primers adding restriction enzyme cleavage sites (indicated above) and cloned and sequenced in pCRII-TOPO. Subsequently, each D34 was subcloned into *proEF1*:*MpTPLNoSTOP* BJ36. All resulting constructs were then subcloned into pSKF HART using *Not*I.

Fusions using the endogenous *MpTPL* promoter were made by excising the *MpTPL* promoter with *Nde*I and *Mlu*I and subcloning it into _*pro*_ *EF1*:*MpTPL-D34* ^*MpIAA*^ BJ36 and subsequently subcloning into pSKF HART using *Not*I.

#### *MpTPL* reporters

Promoter sequences were obtained by assembling partial 454 reads from the first *M*. *polymorpha* genome drafts provided by JGI. Once a putative assembly was made, experimental corroboration of promoters were performed using PCR. A 3.6Kb fragment of the *MpTPL* promoter was amplified using primer MpTPLproF5-NdeI and MpTPLproR1-SalI, and cloned and sequenced in pCRII-TOPO (named 3685). The *MpTPL* promoter was subcloned into *3xVENUSN7BJ36* using *Nde*I and *Sal*I. *3xVENUSN7* has 3 copies of YFP Venus with a C-terminal Nuclear localized sequence [96]. _*pro*_ *MpTPL*:*3xVENUSN7* BJ36 was subcloned into pSKF HART using *Not*I.

#### Cloning of *MpARF1*

*MpARF1* was amplified using RACE with nested PCRs. For 5’RACE, primers UPM, MpARF(miR167)R4-out, NUP and MpARF(miR167)R3-in were used. For 3’RACE, GAGA and ARFmiR167F were used. Full length transcripts were amplified with MpARF1(BamHI)-F1 and MpARF1(EcoRV)-R1 and cloned and sequenced in pCRII-TOPO (Invitrogen). The full-length transcript was subcloned into pE2B using *Bam*HI and *Eco*RV. The resulting plasmid was subcloned into a modified version of pSKF HART with attR1 and attR2 sites that allow a Gateway LR recombination (Invitrogen). The dominant *MpARF1* ^*ΔD34*^ allele was created by assembly PCR, erasing the internal *Not*I site with a silent mutation using Universal M13 Primers and primers MpARF1-mNotI-F2 and MpARF1-mNotI-R2. A second round of PCR using primers MpARF1-ATG-F-KpnI and MpARF1-DeltaD34-R-HindIII removed domains D34 of *MpARF1*. The resulting fragment was sequenced and subcloned into EF1:BJ36 using KpnI/HindIII. All constructs were subcloned into HART binary vector using *Not*I.

#### Cloning of amiRs

amiRs 268 bp in length were designed *in silico* with flanking *Kpn*I and *Hin*dIII sites and synthesized at GeneScript (USA). amiRs were subcloned into EF1:BJ36 using *Kpn*I and *Hin*dIII and later into HART or KART as described in previous sections.

### RLM-RACE

RNA was extracted using QIAGEN Plant RNAeasy Kit. Total RNA was ligated to an RNA oligo (First Choice RLM-RACE) using T4 RNA ligase at 37° for one hour. Samples were precipitated using Phenol:Chlroform and eluates were used as a template for cDNA synthesis using Bioscript Reverse Transcriptase (Bioline), according to manufacturers instructions. PCRs were performed using Ex-Taq (TAKARA) using nested PCR using 5’RACE-Out-F and 5’RACE-in-F (First Choice RLM-RACE) as forward primers. To detect cleaved *MpIAA* transcripts, RLM-MpIAA-R3 and RLM-MpIAA-R1 were used as outer and inner reverse primers, respectively.

### Branching rates measurements

Primary sporeling-transformants expressing either _*pro*_ *EF1*:*MpTPL* ^*N176H*^ or a Hyg^Res^ control T-DNA were simultaneously plated and notches were counted by eye after 25 days of growth in B5 media (N = 17).

### Plant transformation and culture

*M*. *polymorpha* transformation was performed essentially as described previously [92]. *M*. *polymorpha* spores were grown in 25ml liquid 0M51C media for 9 to 11 days prior to *Agrobacterium* co-cultivation. *Agrobacterium* strain GV3001 was transformed with binary vectors and cultured for 2 days at 28°C in LB media whereafter the bacteria was pelleted and cultured for an additional 4 hours in 10ml 0M51C media with 100μM acetorsyringone (Austratech). 500ul bacterial suspension and acetosyringone (100μM final concentration) was added to 25ml spore suspension. Spores and bacteria were co-cultivated for 48–60 hours. Primary transformants (T1 generation) were selected on Gamborgs B5 plates with 10μg/ml Hygromicin (Sigma-Aldrich) and/or G418 (Sigma-Aldrich) and 200μM Timentin (Austratech). After 10–15 days, survivors were re-plated to a second round of selection. After approximately 1 week, survivors were transferred to unsupplemented B5 media or stored at 4°C for later use. Gemmae from the T1 generation (G1 generation) or gemmae from the G1 generation (G2) were used for all experiments, except in cases where no viable gemmae were formed in T1 plants.

### Microscopy

Plants were observed in a Lumar.V12 dissecting microscope (Zeiss) and photographed with an AxioCam HRc (Zeiss). Fixation for SEM was performed in FAA (ethanol 50%, acetic acid 5%, Formaldehyde 10%) overnight at 4°C. Samples were dehydrated in ethanol before critical point drying in a CPD 030 (Baltec). Samples were sputter coated with gold using a SCD 005 (Baltec). Scanning Electron Microscopy was performed with microscope S-570 (Hitachi) at 10kV and photos were digitalized using SPECTRUM Software (Dindima).

## Supporting Information

S1 Fig Effects of exogenous auxin and L-Kyneurenine on thallus growth and development. **(A)** Wild-type gemmaling grown for 18 days on mock (DMSO). **(B)** 18-day-old wild-type gemmaling grown on 10 μM 2,4-D from day six of development. **(C)** 18-day-old wild-type gemmaling grown on auxin from day eight of development. **(D)** 18-day-old wild-type gemmaling grown on auxin from day 12 of development. Arrowheads indicate elongated gemma cups. **(E)** 18-day-old thallus grown on mock. **(F)** 18-day-old thallus grown on 250 μM L-Kyn; fused gemmae cups are observed. **(G)** Wild-type thalli after a month of growth. **(H)**_*pro*_ *MpSHI*:*iaaL* plants fail to elongate laterally, forming narrow thalli after a month of growth. All scale bars are 1 mm, except for A to D, 1cm.
(TIF)

S2 Fig Branching events in *M*. *polymorpha*. Summary of a 20-day-long time-lapse series of wild-type thallus with, initially, a single apical notch (Day 0; white asterisk). By day four, the first gemmae cup is visible (c). By day five, thallus bifurcation can be observed, with two apical notches evident (light blue asterisks). The first gemmae cup has by now been displaced away from the growing point (meristem). The two daughter notches separate by the growth of a mid-lobe (L). By day twelve, new gemmae cups (c’) can be seen behind each of the two daughter notches. By day sixteen, a third branching event is visible by eye and the one new gemmae cups (c’) have matured and start producing gemmae (dark blue asterisks). The asterisks in the central Fig (summary) show the path of a single apical notch following two branching events and 20 days of growth. The path follows the midline of the thallus. The motion through time is due to growth of the thallus tip. Scale bars are 1mm.
(TIF)

S3 Fig Multiple sequence alignment of D34 domains in *M*. *polymorpha*. Alignment shows AUX/IAA as well as class A, B and C ARFs from *Arabidopsis* and *M*. *polymorpha*.
(TIF)

S4 Fig Bayesian phylogeny of the *TOPLESS* gene family across land plants. **(A)** Tree obtained using 23 taxa, 3111 nucleotide characters and ran for 500,000 generations. Numbers above branches indicate posterior probability values. Average standard deviation of split frequencies = 0.007116. Scale bar indicates expected changes/ site.
(TIF)

S5 Fig Bayesian phylogeny of the *AUX/IAA* gene family across land plants. **(A)** Tree obtained using 49 taxa, 309 nucleotide characters and ran for 5,000,000 generations. Numbers above branches indicate posterior probability values. Average standard deviation of split frequencies = 0.008575. Scale bar indicates expected changes/ site.
(TIF)

S6 Fig Unrooted Bayesian phylogeny of ARF genes across land plants. Three distinct clades (classes A, B and C) of ARFs are distinguished, with one *M*. *polymorpha* homolog (red) in each clade. At, *Arabidopsis thaliana* (green); Sm, *Selaginella moellendorffii* (blue); Pp, *Physcomitrella patens* (purple).
(TIF)

S7 Fig Design of amiRs targeting *MpIAA*. Left: Design of two artificial miRNAs targeting the single *MpIAA*. amiRs were designed using the minimal *MpMIR160* stemloop precursor as a backbone and mimicking similar mismatches to generate a miR* sequence. Right: Target sequences for the amiRs used in this study.
(TIF)

S8 Fig Phenotypic variation among _*pro*_ *EF1*:*amiRMpIAA*_*9*_ lines. (**A**) Phenotypes of several independent lines constitutively expressing *amiRMpIAA*_*9*_. **(B)** Semi-quantitative RT-PCR showing transgene (*amiRMpIAA*_*9*_ *)* and full-length target (*MpIAA*) levels in thallus tissues. **(C)** Transcript levels relative to *EF1-alfa* control. Lines with the weakest phenotype (line 1 and 4 as seen in A) have the lowest *amiR* transgene levels. **(D)** Multiple independent _*pro*_ *EF1*:*amiRMpIAA*_*7*_ primary sporeling transformants grown for 14 days in different auxin concentrations show drastic hypersensitivity compared to independent Hyg^Res^ controls. All scale bars, 1 cm.
(TIF)

S9 Fig Sequences of MpARF1 proteins used in this study. Domain 34 is marked in red.
(TIF)

S10 Fig Experiments with MpTPL alleles affect multiple developmental processes. **(A)** Autofluorescence of wild-type gemmalings to illustrate *proMpTPL*:*3XVENUS* lines **(B)** Optical microscopy images of representative *TOPLESS* alleles used in this study. **(C)** Diminished archegoniophore stature in _*pro*_ *EF1*:*MpTPL* ^*N176H*^ plants after a month under Far Red lights. Scale Bars = 1mm.
(TIF)

S11 Fig Nucleotide (A) and protein (B) sequences of MpTPL-D34 fusions. Note how D34^MpARF3^ does not have the MpmiR160 binding site.
(TIF)

S12 Fig Phenotypic effects of MpTPL-D34 chemiric proteins. **(A)**_*pro*_ *EF1*:*MpTPL* plants photographed after 35 days of growth **(B)** Weak _*pro*_ *EF1*:*MpTPL-D34* ^*BDL*^ lines produce a thin thallus that does not extend beyond the midline (similar to _*pro*_ *MpSHI*:*iaaL* lines) and fused cups (similar to _*pro*_ *MpSHI*:*iaaL* and L-Kyn treatment). **(C)** Comparisons between _*pro*_ *EF1*:*MpTPL-D34* ^*MpARF3*^ (left) and _*pro*_ *EF1*:*MpTPL-D34* ^*MpIAA*^ (right) lines show that *MpTPL-D34* ^*MpARF3*^ lines are not as compromised in growth and differentiation as other *MpTPL-D34* fusions. **(D)**_*pro*_ *MpTPL*:*MpTPL-D34* ^*MpIAA*^ lines occasionally produce a single gemmae cup from an aberrant thallus. **(E)**_*pro*_ *MpTPL*:*MpTPL-D34* ^*MpIAA*^ gemmae (*g*) are aberrant and non-symmetrical. Scale bars in A, B, C, D, 1mm; E = 0.5 mm.
(TIF)

S13 Fig Sequences used in this study. *miR160* binding site of *MpARF3* is indicated in red.
(DOCX)

S1 Table D34 protein sequence identity based on EMBOSS Matcher pairwise alignment. (DOC)

S2 Table _*pro*_ *EF1*:*MpTPL* ^*N176H*^ results in additional branching events. (DOC)

S3 Table Notch and gemmae cup production in mature MpARF1 overexpressing lines. Numbers of notches and cups represent averages over the 30 lines.
(DOC)

S4 Table Summary of phenotypic effects of constructs in this work. (DOC)

S5 Table Primers used in this study. (DOC)
