# Correction: A Simple Auxin Transcriptional Response System Regulates Multiple Morphogenetic Processes in the Liverwort *Marchantia polymorpha*

**DOI:** 10.1371/journal.pgen.1005900

**Published:** 2016-02-22

**Authors:** Eduardo Flores-Sandoval, D. Magnus Eklund, John L. Bowman

Errors were made during the preparation of this manuscript that deviate from accepted scientific norms. Specifically, (1) one figure is flipped horizontally in the control panel of two figures representing the same experiment (Fig 1E and S1A); (2) the same photograph of a genotype is used in Fig 1O that is presented as a control genotype in Fig 4G of a manuscript published in a different journal (Eklund, D.M., Ishizaki, K., Flores-Sandoval, E., Kikuchi, S., Takebayashi, Y., Tsukamoto, S., Hirakawa, Y., Nonomura, M., Kato, H., Kouno, M., Bhalerao, R.P., Lagercrantz, U., Kasahara, H., Kohchi, T., and Bowman, J.L. (2015). Auxin produced by the indole-3-pyruvic acid pathway regulates development and gemmae dormancy in the liverwort Marchantia polymorpha. Plant Cell 27: 1650–1669.); a corrigendum has been published for the latter paper; and (3) in the reorganization of the manuscript from the initial submission into its final form, Fig 2B was mislabeled, giving the appearance of using the same control figures to represent different experiments (Fig 2B and S8D Fig).

**Fig 1:**
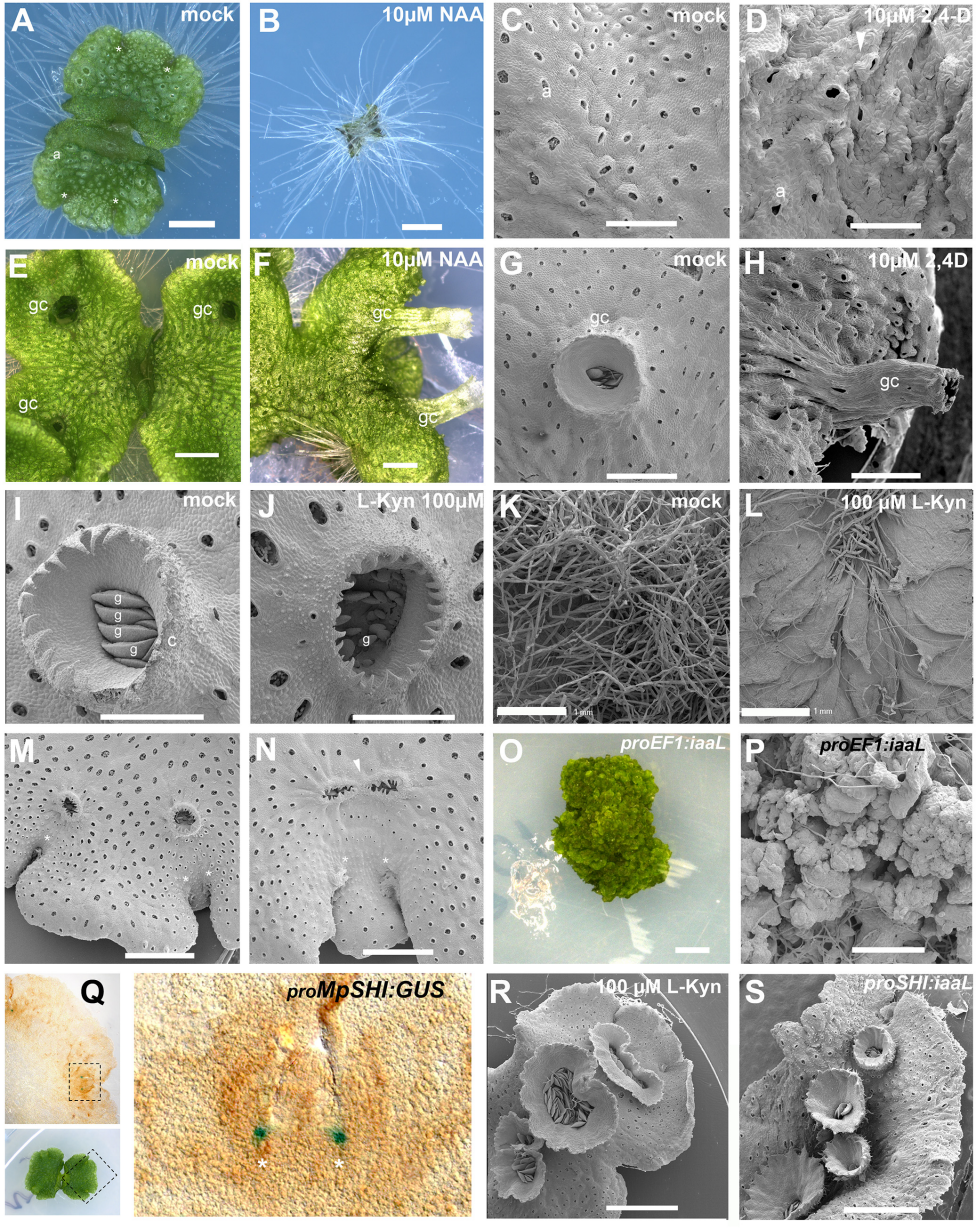
Developmental effects of auxin in the *M*. *polymorpha* thallus. **(A)** 10-day-old wild-type gemmaling with four apical notches and well developed air pores. **(B)** 10-day-old wild-type gemmae grown on 10 μM NAA. **(C)** Dorsal epidermis of 15-day-old wild-type thallus. **(D)** Dorsal epidermis of 15-day-old wild-type thallus, transferred on day 6 to 10 μM 2,4-D with abnormal air pores. **(E)** 18-day-old wild-type thallus with three gemmae cups. **(F)** 23-day-old wild-type thallus transferred at day 16 to 10 μM NAA with elongated gemmae cup. **(G)** Wild-type mature gemmae cup. **(H)** Wild-type mature gemmae cup from 17-day-old plant, transferred at day 12 to 10 μM 2,4-D. **(I)** Wild-type mature gemmae cup with gemmae inside. **(J)** Wild-type mature gemmae cup from a plant grown on 100 μM L-Kyn. **(K)** Ventral side of wild-type thallus grown on mock media. **(L)** Ventral side of wild-type thallus grown on 100 μM L-Kyn; a decrease in rhizoid number exposes ventral scales. **(M)** Mature thallus showing a thallus lobe separating two recently bifurcated apical notches. **(N)** Mature thallus of plants transferred on day 7 to 100 μM L-Kyn, showing a smaller lobe spacing apical notches and fused gemmae cup primordia (arrowhead). **(O)** Strong _*pro*_*EF1*:*iaaL* lines are composed of an undifferentiated mass of cells that fail to establish a clear dorsiventrality. **(P)** Close up view of _*pro*_*EF1*:*iaaL* line shows a mass of undifferentiated cells although rhizoids are able to differentiate. **(Q)** Expression pattern of _*pro*_*MpSHI* in mature thallus with staining in the vicinity of the apical cell. **(R)** Different degrees of gemmae cup fusion observed in 100 μM L-Kyn treated plants. **(S)**_*pro*_*MpSHI*:*iaaL* plants show an irregular zig-zag arrangement of gemmae cups that are in close proximity to each other. Scale bars in A, B, E, F, G, H, J, K, L, O, R and S = 1 mm; C, D and P = 0.6 mm; I = 0.5 mm; M = 1.2 mm; N = 0.75 mm; Q = 0.2 mm. Asterisk, apical notch; a, air pores; gc, gemma cups; g, gemmae.

**Fig 2:**
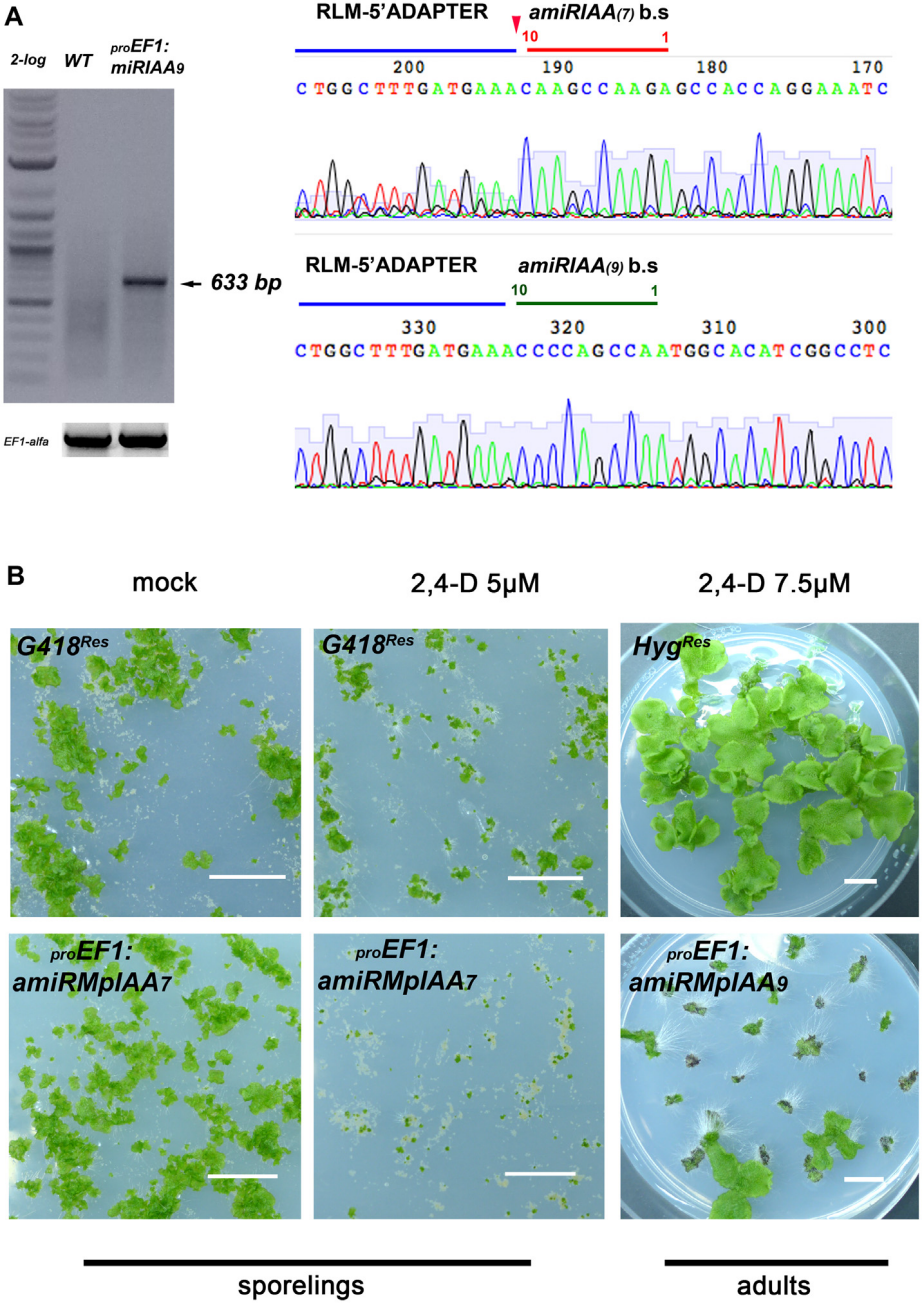
*MpIAA* represses auxin signaling in *M*. *polymorpha*. **(A)** RLM RACE of *amiRMpIAA* expressing lines. Left panel shows gel electrophoresis of RLM-RACE products. Right panel shows direct sequencing of the 633bp RLM-RACE product from *amiRMpIAA*_*9*_. Sequencing detected the expected cleavage of *MpIAA* between bases 10/11 of both miR sequences. **(B)** Auxin hypersensitivity in knock-down *MpIAA* lines at both the sporeling and adult stages. At the sporeling stage, _*pro*_*EF1*:*amiRMpIAA*_*7*_ sporelings grown on 5μM 2,4-D from germination have severely affected area compared to *G418*^*Res*^ controls. In the adult stage plants were grown for 16 days on B5 media prior to treatment with 2,4-D. _*pro*_*EF1*:*amiRMpIAA*_*9*_ treated with 7.5μM 2,4-D for ten days form ectopic rhizoids compared to *Hyg*^*Res*^ controls. In all panels, each of the plants is an independent transformant. Scale Bars = 1 cm.

The authors provide the correct versions of Figs 1 and 2 and S8 Fig here.

The subsection ‘Cloning of amiRs.’ in the Materials and Methods section should read:

Cloning of amiRs. amiRs 268 bp in length were designed *in silico* with flanking *Kpn*I and *Hin*dIII sites and synthesized at GeneScript (USA). amiRs were subcloned into EF1:BJ36 using *Kpn*I and *Hin*dIII and later into binary vectors using NotI/ScaI and blue white selection. *amiRMpIAA*_*9*_ was cloned into HART (a binary vector conferring Hygromicin Resistance in plants) and *amiRMpIAA*_*7*_ was cloned into KART (Binary Vector conferring G418 Resistance).

## Supporting Information

S8 Fig Phenotypic variation among proEF1:amiRMpIAA9 lines. **(A)** Phenotypes of several independent lines constitutively expressing amiRMpIAA9. **(B)** Semi-quantitative RT-PCR showing transgene (amiRMpIAA9) and full-length target (MpIAA) levels in thallus tissues. **(C)** Transcript levels relative to EF1-alfa control. Lines with the weakest phenotype (line 1 and 4 as seen in A) have the lowest amiR transgene levels.
(JPG)
