# Supplementary figures and images for: A Simple Auxin Transcriptional Response System Regulates Multiple Morphogenetic Processes in the Liverwort Marchantia polymorpha
Source: PLoS Genet. 2015 May 28;11(5):e1005207. doi: 10.1371/journal.pgen.1005207 (PMC4447368; doi:10.1371/journal.pgen.1005207)

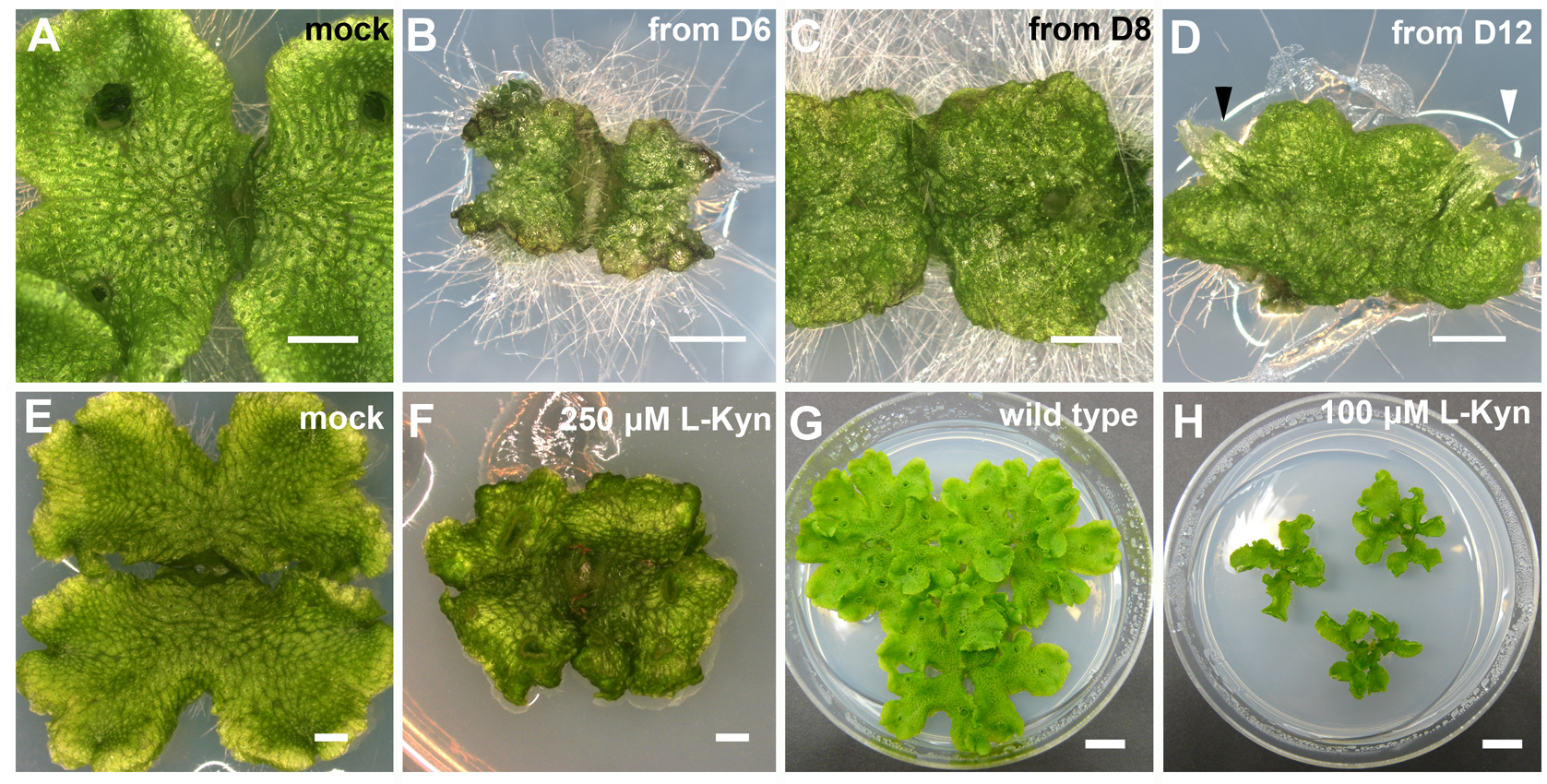

Supplement: S1 Fig — (A) Wild-type gemmaling grown for 18 days on mock (DMSO). (B) 18-day-old wild-type gemmaling grown on 10 μM 2,4-D from day six of development. (C) 18-day-old wild-type gemmaling grown on auxin from day eight of development. (D) 18-day-old wild-type gemmaling grown on auxin from day 12 of development. Arrowheads indicate elongated gemma cups. (E) 18-day-old thallus grown on mock. (F) 18-day-old thallus grown on 250 μM L-Kyn; fused gemmae cups are observed. (G) Wild-type thalli after a month of growth. (H) pro MpSHI:iaaL plants fail to elongate laterally, forming narrow thalli after a month of growth. All scale bars are 1 mm, except for A to D, 1cm. (TIF) [file pgen.1005207.s001.tif]

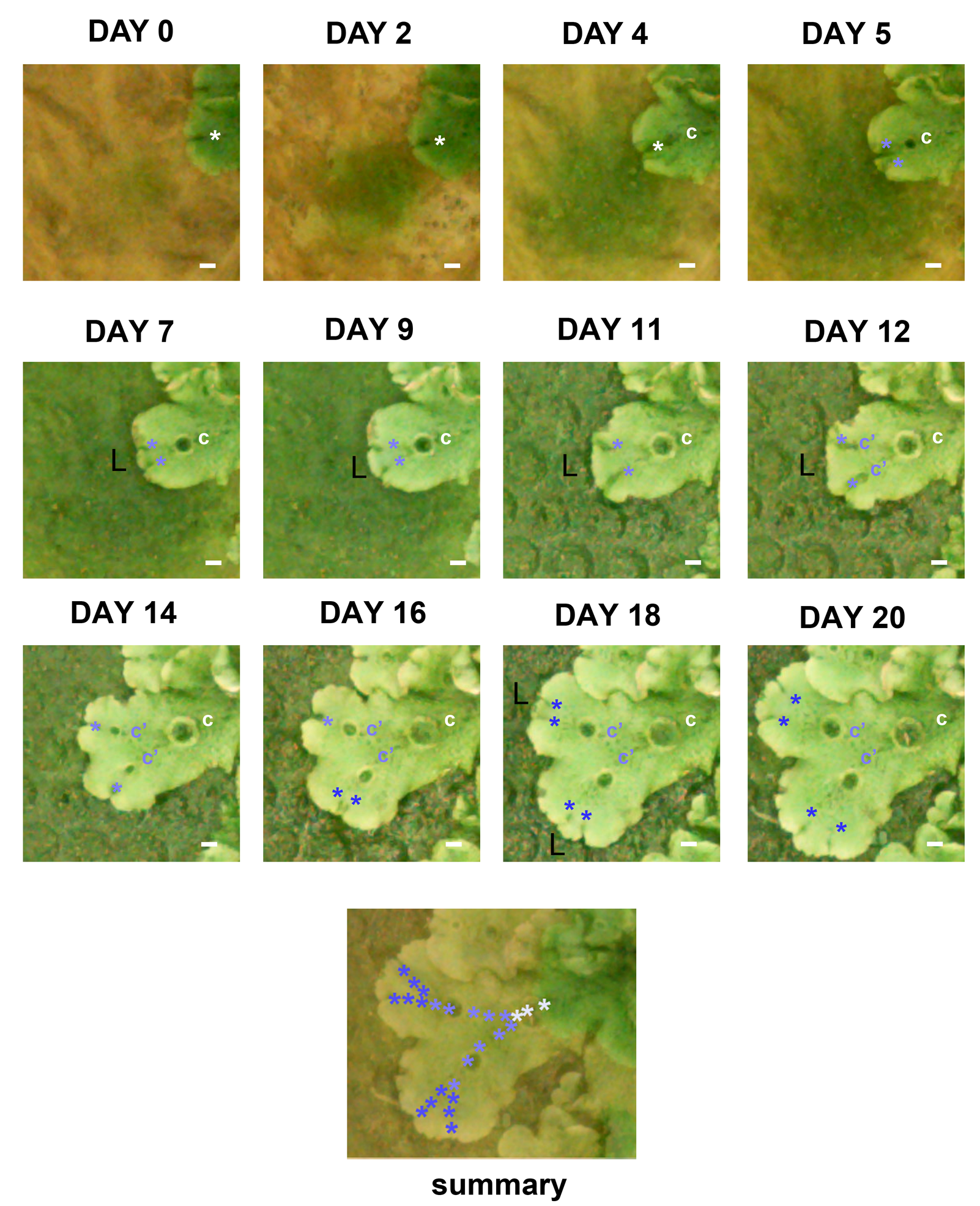

Supplement: S2 Fig — Summary of a 20-day-long time-lapse series of wild-type thallus with, initially, a single apical notch (Day 0; white asterisk). By day four, the first gemmae cup is visible (c). By day five, thallus bifurcation can be observed, with two apical notches evident (light blue asterisks). The first gemmae cup has by now been displaced away from the growing point (meristem). The two daughter notches separate by the growth of a mid-lobe (L). By day twelve, new gemmae cups (c’) can be seen behind each of the two daughter notches. By day sixteen, a third branching event is visible by eye and the one new gemmae cups (c’) have matured and start producing gemmae (dark blue asterisks). The asterisks in the central Fig (summary) show the path of a single apical notch following two branching events and 20 days of growth. The path follows the midline of the thallus. The motion through time is due to growth of the thallus tip. Scale bars are 1mm. (TIF) [file pgen.1005207.s002.tif]

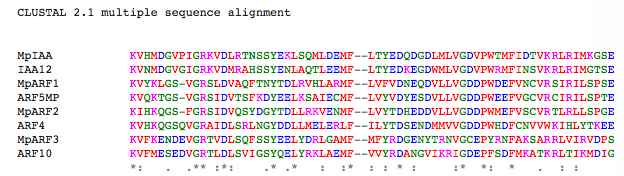

Supplement: S3 Fig — Alignment shows AUX/IAA as well as class A, B and C ARFs from Arabidopsis and M. polymorpha. (TIF) [file pgen.1005207.s003.tif]

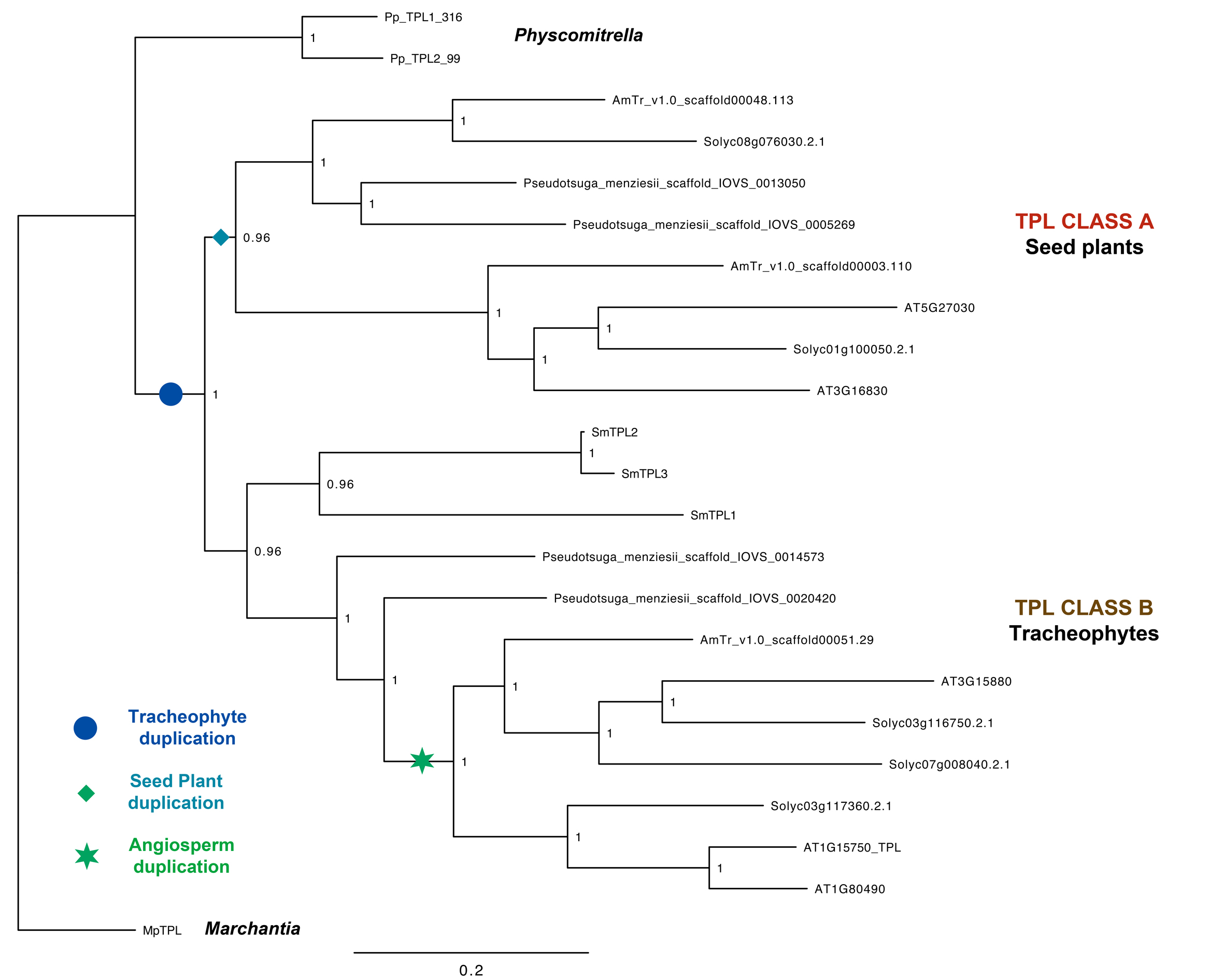

Supplement: S4 Fig — (A) Tree obtained using 23 taxa, 3111 nucleotide characters and ran for 500,000 generations. Numbers above branches indicate posterior probability values. Average standard deviation of split frequencies = 0.007116. Scale bar indicates expected changes/ site. (TIF) [file pgen.1005207.s004.tif]

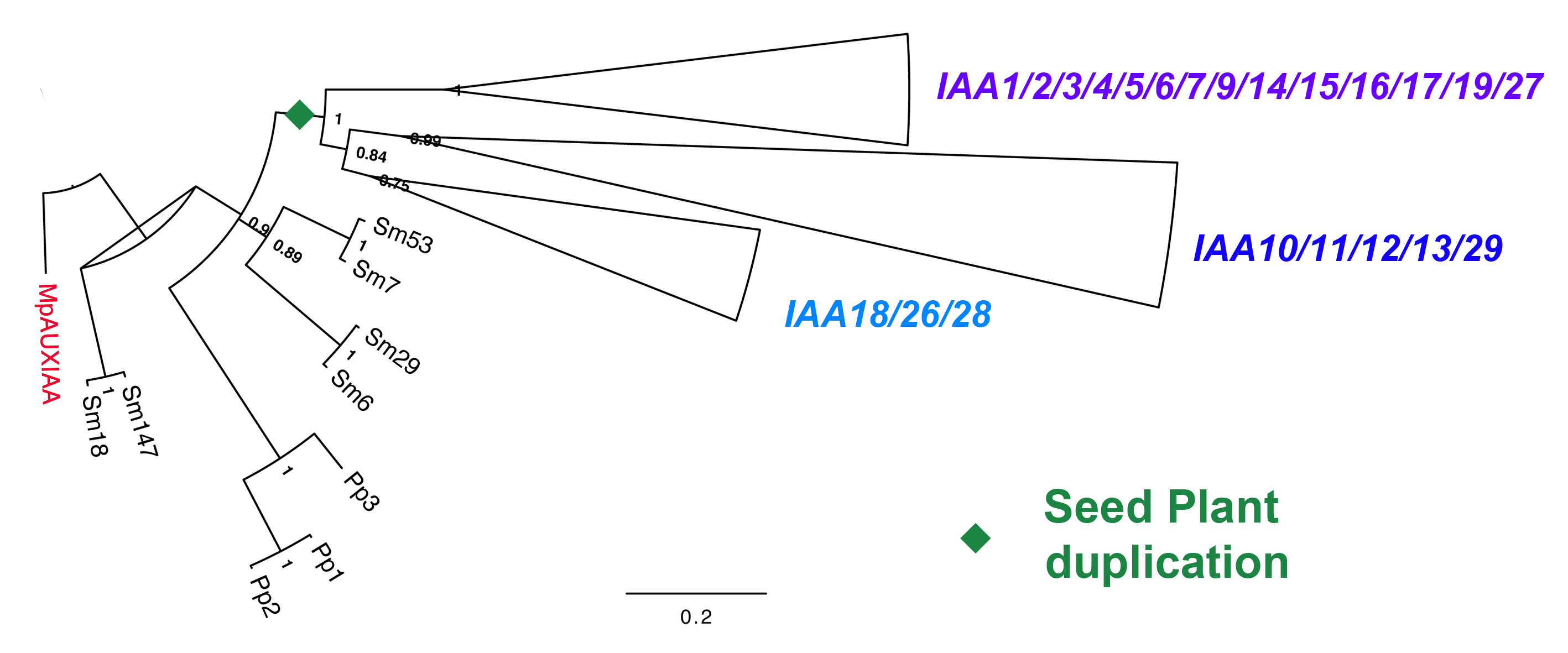

Supplement: S5 Fig — (A) Tree obtained using 49 taxa, 309 nucleotide characters and ran for 5,000,000 generations. Numbers above branches indicate posterior probability values. Average standard deviation of split frequencies = 0.008575. Scale bar indicates expected changes/ site. (TIF) [file pgen.1005207.s005.tif]

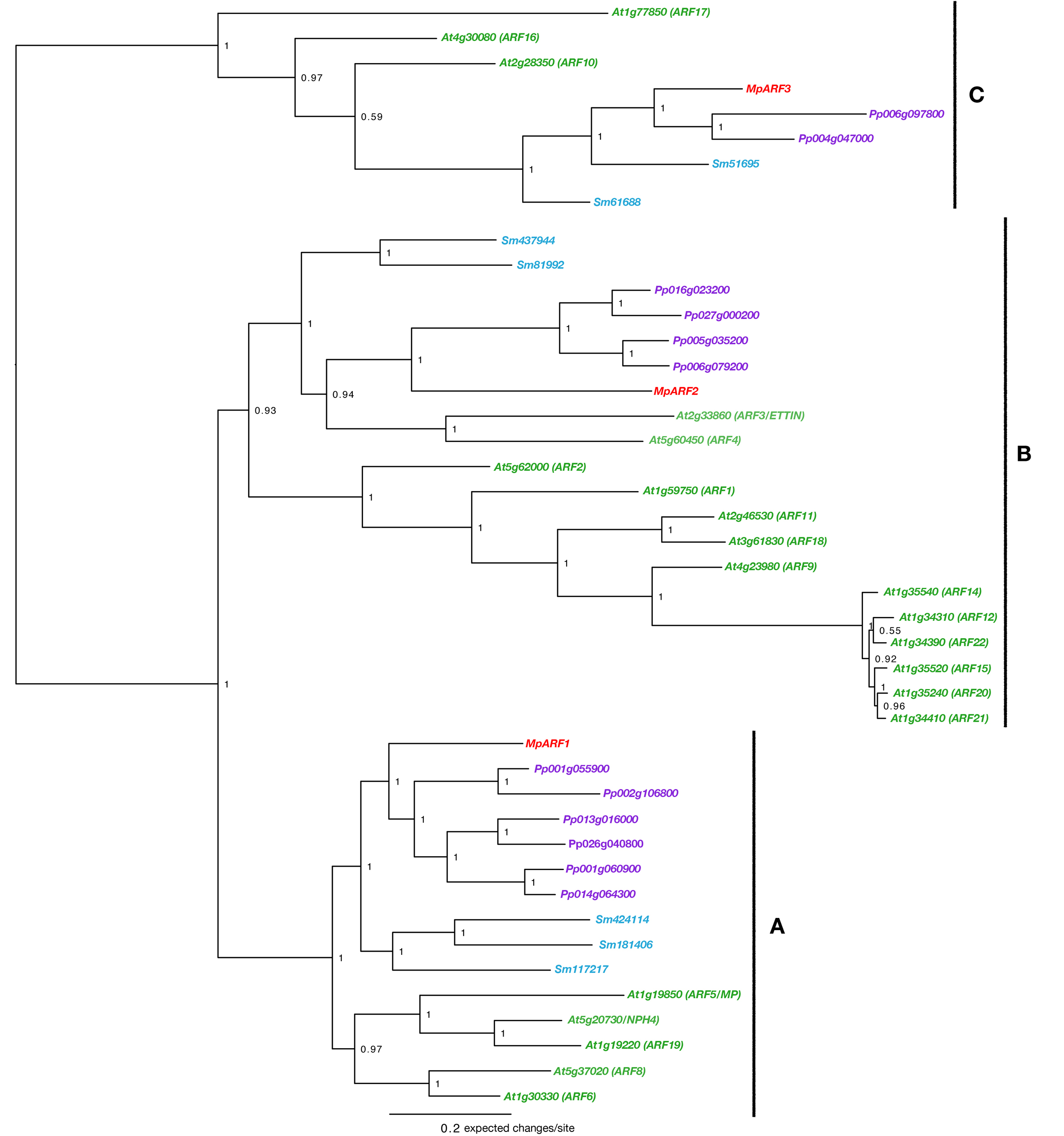

Supplement: S6 Fig — Three distinct clades (classes A, B and C) of ARFs are distinguished, with one M. polymorpha homolog (red) in each clade. At, Arabidopsis thaliana (green); Sm, Selaginella moellendorffii (blue); Pp, Physcomitrella patens (purple). (TIF) [file pgen.1005207.s006.tif]

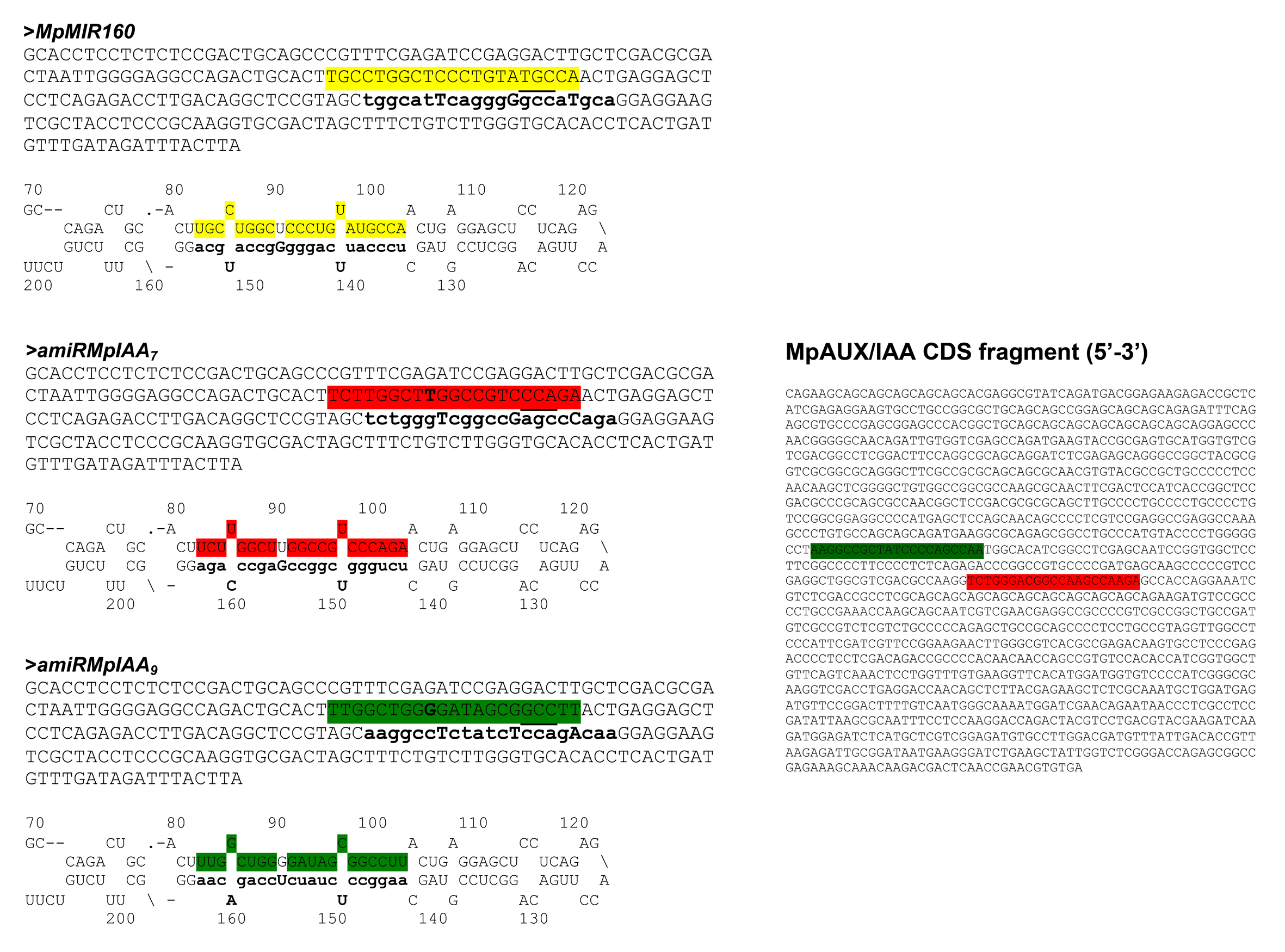

Supplement: S7 Fig — Left: Design of two artificial miRNAs targeting the single MpIAA. amiRs were designed using the minimal MpMIR160 stemloop precursor as a backbone and mimicking similar mismatches to generate a miR* sequence. Right: Target sequences for the amiRs used in this study. (TIF) [file pgen.1005207.s007.tif]

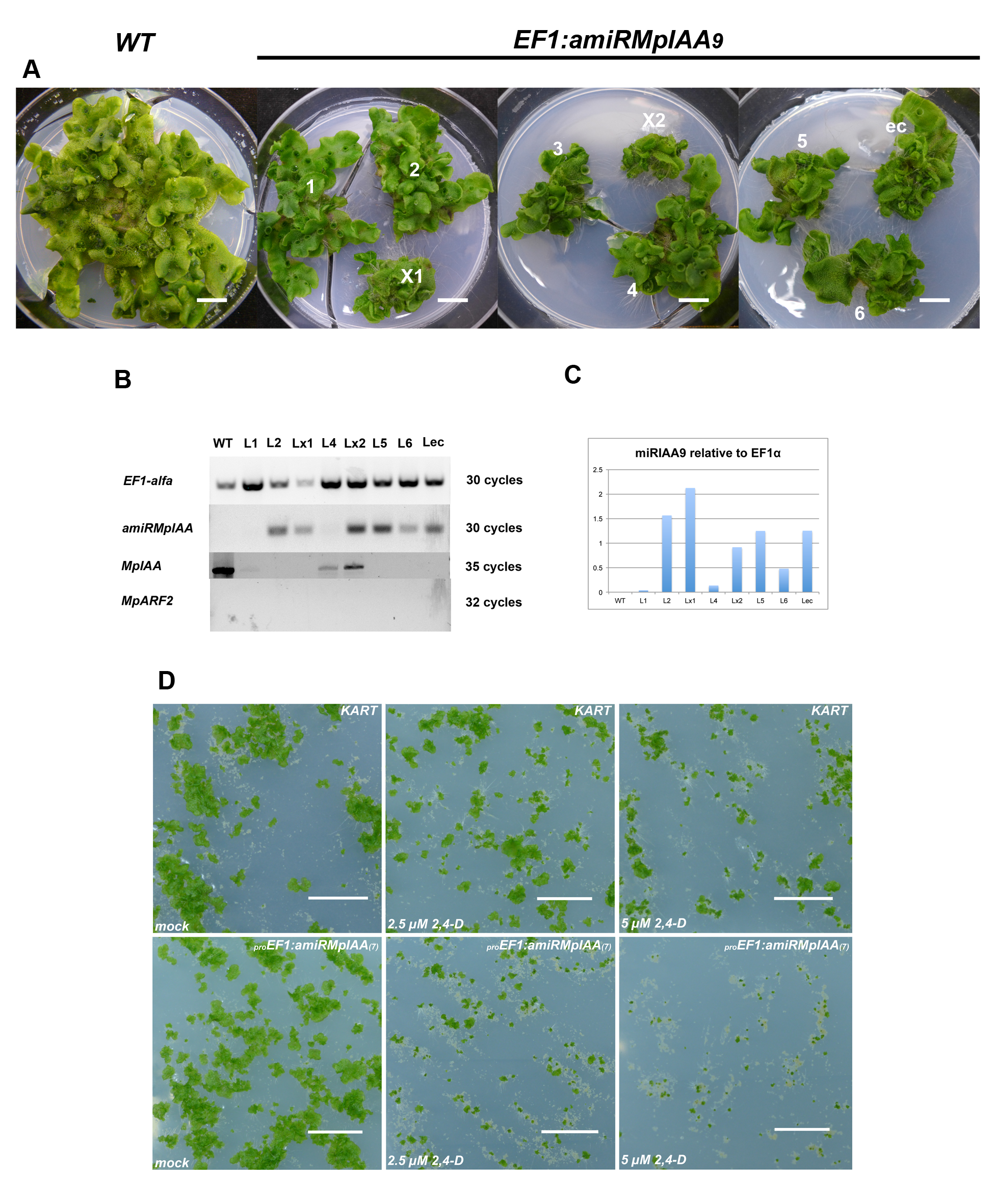

Supplement: S8 Fig — (A) Phenotypes of several independent lines constitutively expressing amiRMpIAA 9. (B) Semi-quantitative RT-PCR showing transgene (amiRMpIAA 9 ) and full-length target (MpIAA) levels in thallus tissues. (C) Transcript levels relative to EF1-alfa control. Lines with the weakest phenotype (line 1 and 4 as seen in A) have the lowest amiR transgene levels. (D) Multiple independent pro EF1:amiRMpIAA 7 primary sporeling transformants grown for 14 days in different auxin concentrations show drastic hypersensitivity compared to independent HygRes controls. All scale bars, 1 cm. (TIF) [file pgen.1005207.s008.tif]

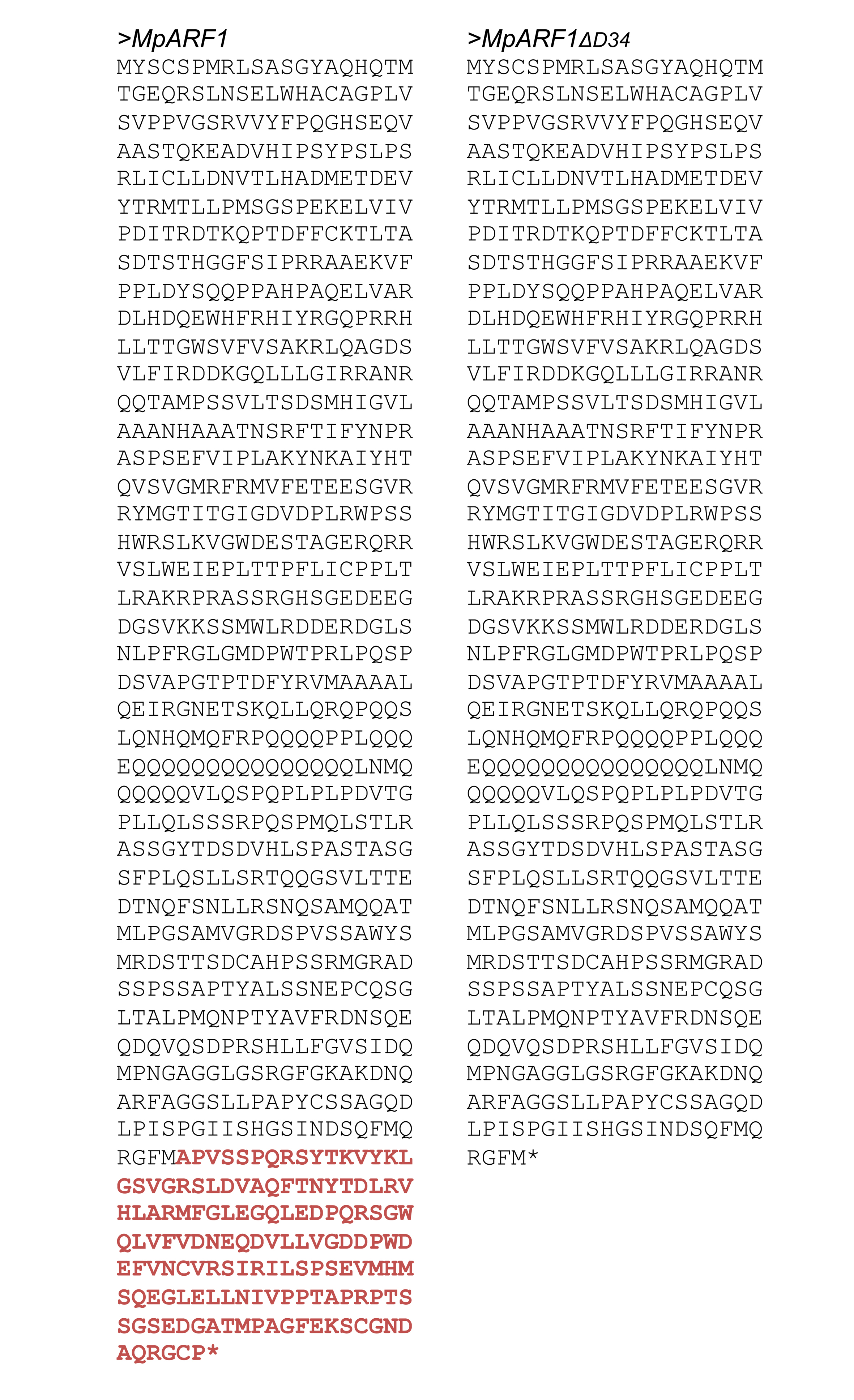

Supplement: S9 Fig — Domain 34 is marked in red. (TIF) [file pgen.1005207.s009.tif]

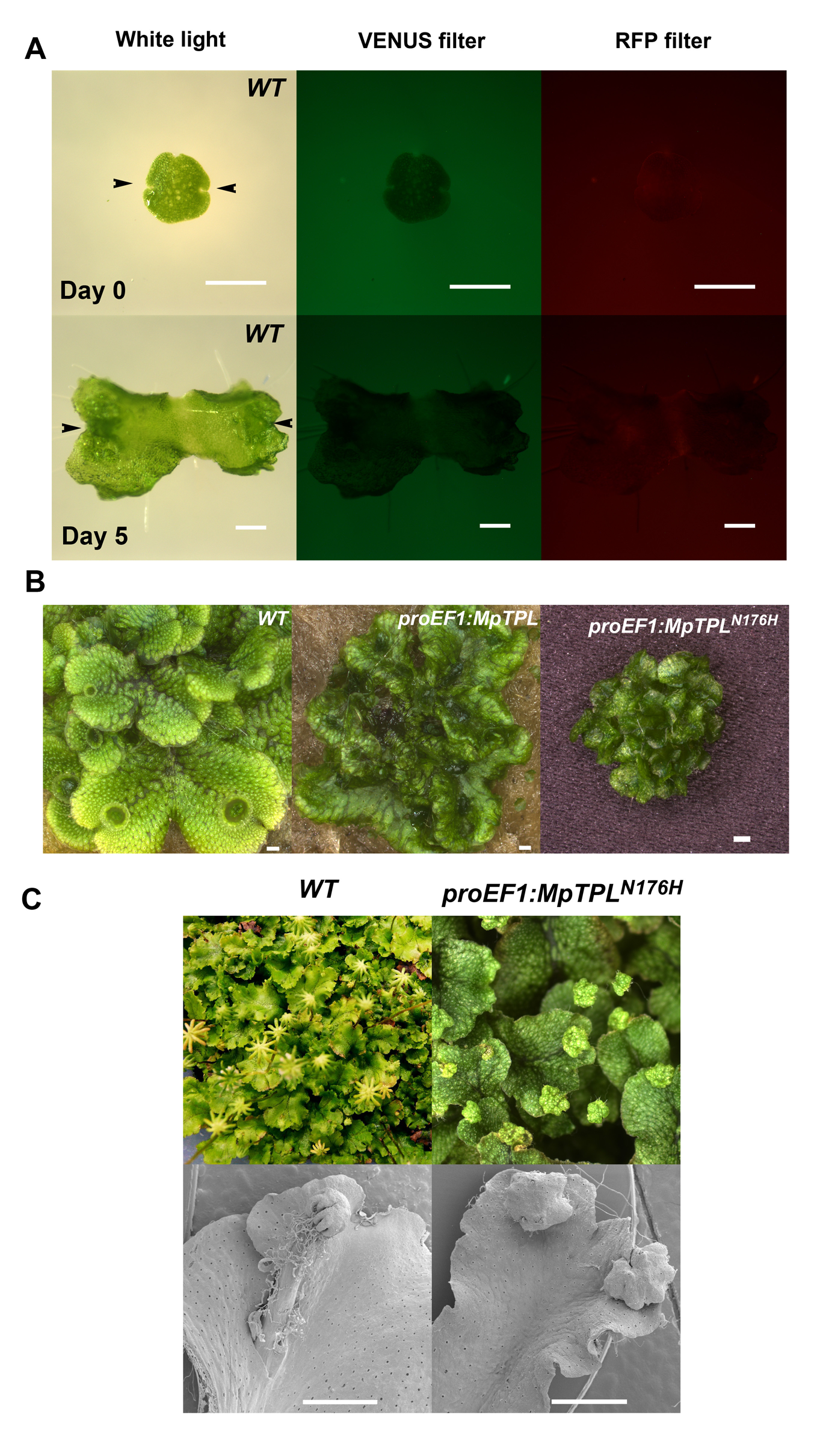

Supplement: S10 Fig — (A) Autofluorescence of wild-type gemmalings to illustrate proMpTPL:3XVENUS lines (B) Optical microscopy images of representative TOPLESS alleles used in this study. (C) Diminished archegoniophore stature in pro EF1:MpTPL N176H plants after a month under Far Red lights. Scale Bars = 1mm. (TIF) [file pgen.1005207.s010.tif]

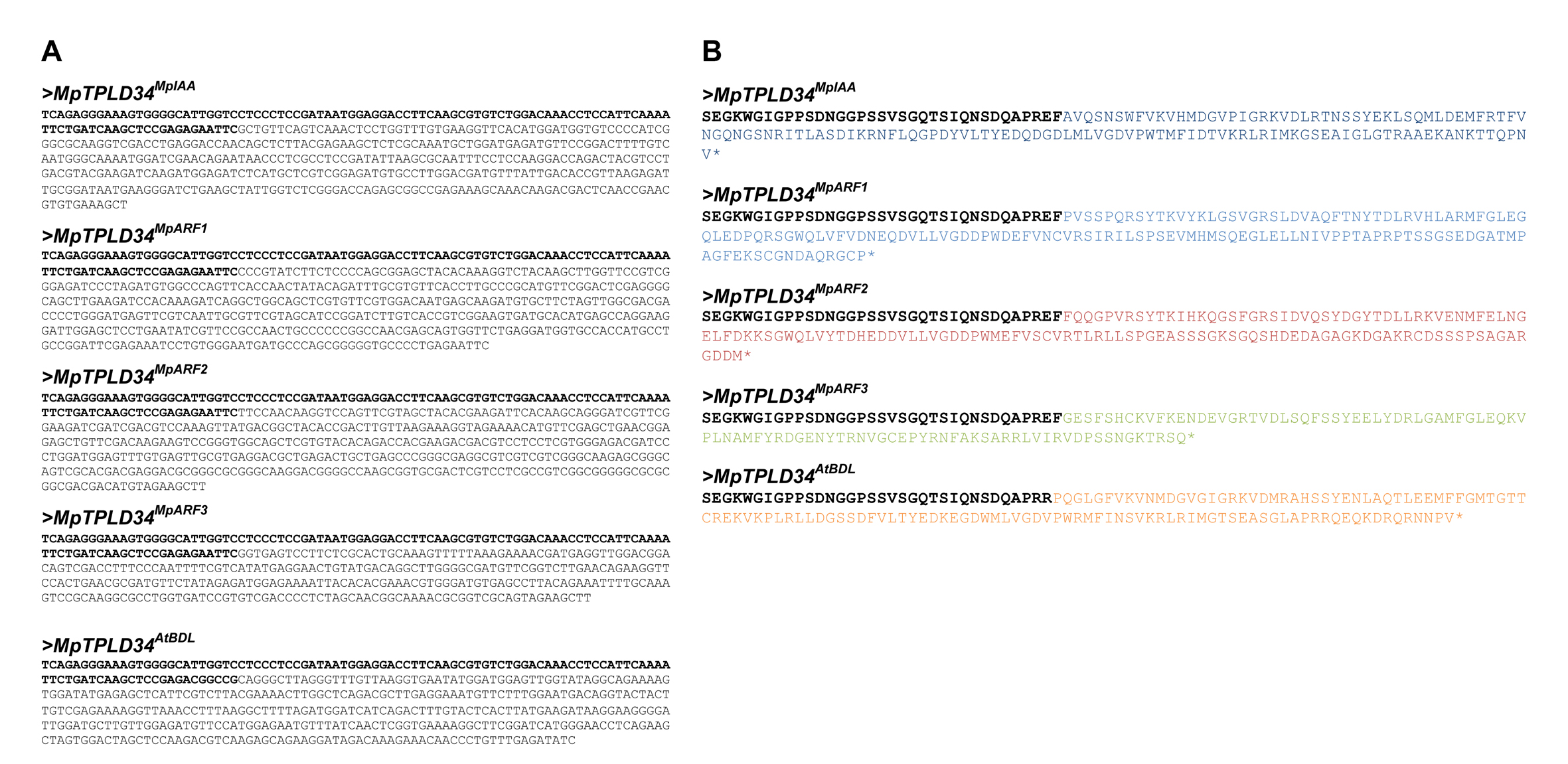

Supplement: S11 Fig — Note how D34MpARF3 does not have the MpmiR160 binding site. (TIF) [file pgen.1005207.s011.tif]

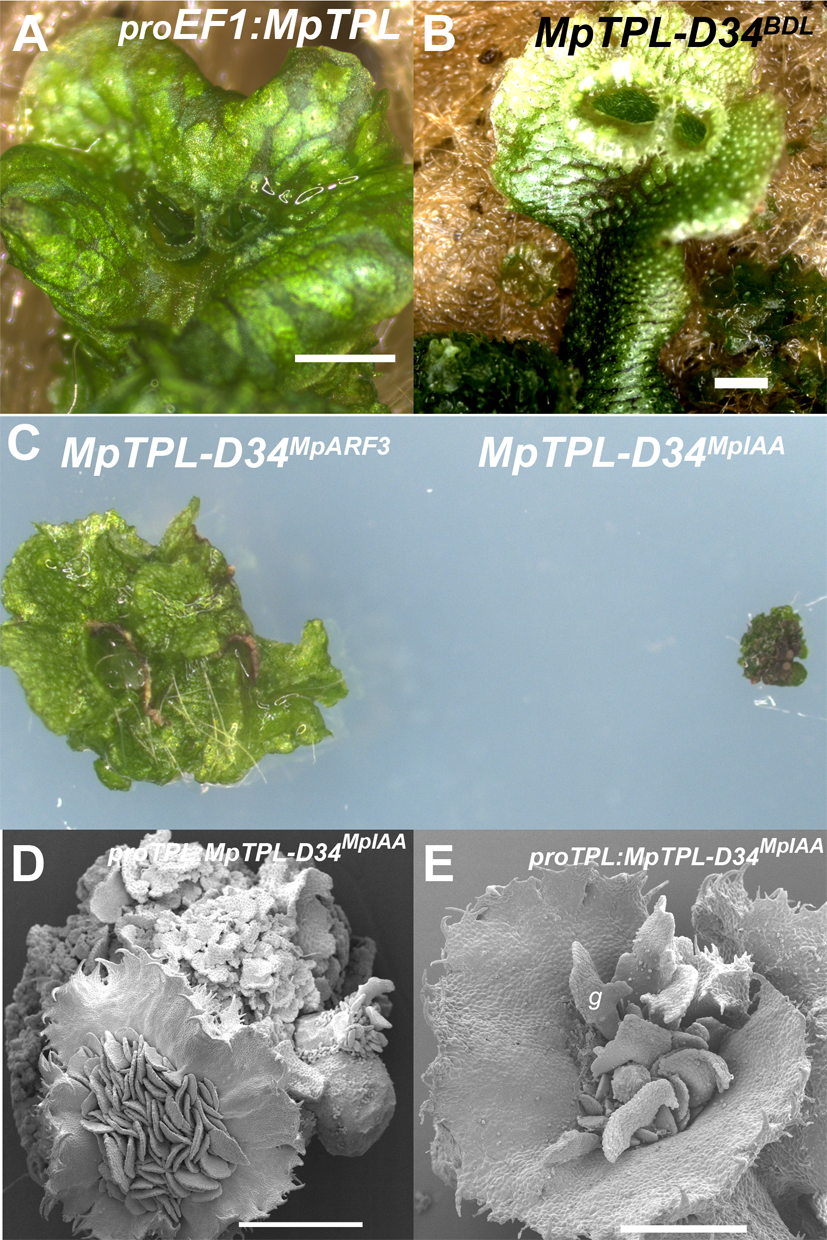

Supplement: S12 Fig — (A) pro EF1:MpTPL plants photographed after 35 days of growth (B) Weak pro EF1:MpTPL-D34 BDL lines produce a thin thallus that does not extend beyond the midline (similar to pro MpSHI:iaaL lines) and fused cups (similar to pro MpSHI:iaaL and L-Kyn treatment). (C) Comparisons between pro EF1:MpTPL-D34 MpARF3 (left) and pro EF1:MpTPL-D34 MpIAA (right) lines show that MpTPL-D34 MpARF3 lines are not as compromised in growth and differentiation as other MpTPL-D34 fusions. (D) pro MpTPL:MpTPL-D34 MpIAA lines occasionally produce a single gemmae cup from an aberrant thallus. (E) pro MpTPL:MpTPL-D34 MpIAA gemmae (g) are aberrant and non-symmetrical. Scale bars in A, B, C, D, 1mm; E = 0.5 mm. (TIF) [file pgen.1005207.s012.tif]
