# Supplementary material for: A Simple Auxin Transcriptional Response System Regulates Multiple Morphogenetic Processes in the Liverwort Marchantia polymorpha
Source: PLoS Genet. 2015 May 28;11(5):e1005207. doi: 10.1371/journal.pgen.1005207 (PMC4447368; doi:10.1371/journal.pgen.1005207)
Supplement: S13 Fig — miR160 binding site of MpARF3 is indicated in red. (DOCX) [file pgen.1005207.s013.docx]

**>MpIAA**

ATGAGTCAAAATGCGCATGCTGCCGTGCTTAGTCGAGGTTCAGGTGCAGGGGGAGCGATTGGGTTAGGTCACAGTGTTAGTAATCCCCCGGGGAGTAGCAGCAACATGAATCTGAGCTCTGCGTCCTCCGTGTCGTGGAGTCTTGTGAACAATGGCGCTCTGACATCTGGAGCTTCCGGAAACCAGAACATTTCGGCCTCCGCCAGCCATCAGCAGCAGCAGCAGGATGCCAAGGCTGCTGCTTCTCCGCAGAACAGCAATCAGAGCGGCTCCACGCTGAAGGAGCACGACCTCCTAGGCTTATCGGAGGTCTCGTCCTCGACCAGCCGAGGAAGCCCCGTCACCCAGGACAGTCTGCAGGACGAGTGCAATGATTTCGAGGAGTTAAATCTGAAATTGGGTCCTCCAGCAGTCCGCAAGAACTCGTACCCAAGAACTCAAGCCTCGGAGCCGCAGCAGCAGCAAGCTCCGGCAGTGCTCGAAAGTGGAAACTCCCACGCAGGCTCGGAGGTGAGCCAGCAAGCCACGGAGCAGGGGACGGCCTTCGCGGAGAAGAGCCCCTCGAGCGCAGAACAGAACGCCGCAGCCAACGAGCAGCAGCAGCAGCAACAGGCCGATCGAGGCCTGGCCAGCTATGGCGTGCGCGGGGGTGGCGACATGTCCCAAGCGGGGATGGCCGATGCCGCCTCGGGCAACCAGGGCTGCCGACCCTCCATCGACAGCGAGGCCCTTATGAAATGGCACGCCGAGCAAGTCGGCGATCGCAAGACGTCGAACGAGTCTCTCCGACTCGGAATCTCTCTGCACTCGCAGCAGCGCGGGCCGGCGCAGGATGGAGTCAGCGAGAAGAAGCCATTTCTCAGCTCCGTCGACCCCAAACAGCAACAGCAGCAGCAGCAACCACAGCAAGACGATCCATGTTGGCAGGCAGACCGCAATTTCCAAGACGACGTCTCGAACATGGACAACAACATCATCAGCAACAAGCAAGAACGCCCCTCCGTGGTCCAGGAGCAGCAGCAACAACAACAACAGCAGCAGGTCGCCAAGCAACAACAGCAGCAGCAGCAGCACCTCGAGAGGCAGAAGCAGCAGCAGCAGCAGCACGAGGCGTATCAGATGACGGAGAAGAGACCGCTCATCGAGAGGAAGTGCCTGCCGGCGCTGCAGCAGCCGGAGCAGCAGCAGAGATTTCAGAGCGTGCCCGAGCGGAGCCCACGGCTGCAGCAGCAGCAGCAGCAGCAGCAGGAGCCCAACGGGGGCAACAGATTGTGGTCGAGCCAGATGAAGTACCGCGAGTGCATGGTGTCGTCGACGGCCTCGGACTTCCAGGCGCAGCAGGATCTCGAGAGCAGGGCCGGCTACGCGGTCGCGGCGCAGGGCTTCGCCGCGCAGCAGCGCAACGTGTACGCCGCTGCCCCCTCCAACAAGCTCGGGGCTGTGGCCGGCGCCAAGCGCAACTTCGACTCCATCACCGGCTCCGACGCCCGCAGCGCCAACGGCTCCGACGCGCGCAGCTTGCCCCTGCCCCTGCCCCTGTCCGGCGGAGGCCCCATGAGCTCCAGCAACAGCCCCTCGTCCGAGGCCGAGGCCAAAGCCCTGTGCCAGCAGCAGATGAAGGCGCAGAGCGGCCTGCCCATGTACCCCTGGGGGCCTAAGGCCGCTATCCCCAGCCAATGGCACATCGGCCTCGAGCAATCCGGTGGCTCCTTCGGCCCCTTCCCCTCTCAGAGACCCGGCCGTGCCCCGATGAGCAAGCCCCCGTCCGAGGCTGGCGTCGACGCCAAGGTCTGGGACGGCCAAGCCAAGAGCCACCAGGAAATCGTCTCGACCGCCTCGCAGCAGCAGCAGCAGCAGCAGCAGCAGCAGAAGATGTCCGCCCCTGCCGAAACCAAGCAGCAATCGTCGAACGAGGCCGCCCCGTCGCCGGCTGCCGATGTCGCCGTCTCGTCTGCCCCCAGAGCTGCCGCAGCCCCTCCTGCCGTAGGTTGGCCTCCCATTCGATCGTTCCGGAAGAACTTGGGCGTCACGCCGAGACAAGTGCCTCCCGAGACCCCTCCTCGACAGACCGCCCCACAACAACCAGCCGTGTCCACACCATCGGTGGCTGTTCAGTCAAACTCCTGGTTTGTGAAGGTTCACATGGATGGTGTCCCCATCGGGCGCAAGGTCGACCTGAGGACCAACAGCTCTTACGAGAAGCTCTCGCAAATGCTGGATGAGATGTTCCGGACTTTTGTCAATGGGCAAAATGGATCGAACAGAATAACCCTCGCCTCCGATATTAAGCGCAATTTCCTCCAAGGACCAGACTACGTCCTGACGTACGAAGATCAAGATGGAGATCTCATGCTCGTCGGAGATGTGCCTTGGACGATGTTTATTGACACCGTTAAGAGATTGCGGATAATGAAGGGATCTGAAGCTATTGGTCTCGGGACCAGAGCGGCCGAGAAAGCAAACAAGACGACTCAACCGAACGTGTGA

**>MpARF1**

ATGTATTCTTGTTCGCCGATGAGGCTGTCTGCCTCTGGATATGCGCAACATCAAACAATGACAGGTGAACAGCGAAGTCTTAACTCTGAGCTATGGCATGCATGTGCGGGTCCTCTTGTGTCTGTGCCTCCCGTGGGCAGCCGCGTGGTCTACTTTCCTCAGGGTCATAGCGAGCAGGTGGCTGCATCAACCCAAAAGGAGGCCGATGTGCATATACCCAGCTATCCCAGTCTCCCATCGCGGTTGATCTGTTTGCTCGACAATGTTACCCTCCATGCGGATATGGAGACGGATGAAGTTTACACCCGGATGACACTGCTACCTATGAGTGGGAGTCCGGAGAAGGAACTTGTGATCGTGCCTGACATCACAAGGGACACCAAACAACCTACAGATTTTTTCTGCAAAACATTGACAGCAAGTGATACTAGTACTCATGGTGGCTTTTCAATTCCCCGCCGTGCCGCAGAGAAAGTCTTTCCACCCCTGGACTACTCCCAACAACCTCCAGCCCATCCTGCCCAGGAGCTTGTCGCAAGAGACCTTCATGATCAGGAGTGGCACTTCCGGCACATCTACAGAGGTCAACCTCGTAGACATCTTCTCACAACTGGATGGAGTGTTTTTGTTAGTGCTAAGAGACTCCAGGCTGGTGATTCTGTTCTTTTTATCAGAGATGACAAGGGACAGCTTCTTCTAGGCATAAGGCGTGCTAACAGGCAGCAGACAGCAATGCCTTCCTCAGTGCTCACGAGTGACAGCATGCATATTGGTGTTCTTGCGGCCGCTAACCATGCAGCCGCCACCAATAGCAGATTCACTATCTTTTACAATCCCAGGGCAAGCCCATCTGAGTTCGTAATCCCTCTTGCTAAGTACAATAAAGCTATCTACCACACCCAGGTTTCTGTCGGGATGCGTTTCAGGATGGTATTTGAAACAGAGGAATCAGGTGTGCGAAGATACATGGGAACTATTACTGGTATCGGCGATGTCGACCCGCTTAGATGGCCGAGCTCGCACTGGCGGTCGTTGAAGGTTGGCTGGGATGAATCTACAGCCGGCGAGAGACAGAGGCGTGTGTCTCTGTGGGAAATTGAGCCGTTAACAACCCCTTTCCTGATCTGTCCTCCACTGACATTGAGAGCGAAGCGGCCCCGGGCGTCTTCTCGAGGCCATTCGGGTGAAGATGAGGAAGGAGATGGCTCGGTCAAGAAGTCTTCTATGTGGTTAAGAGATGACGAGAGAGACGGTCTATCGAATCTACCTTTCAGGGGCCTTGGGATGGACCATTGGACACCCAGGTTACCACAGAGTCCAGATTCAGTAGCTCCTGGTACACCAACAGATTTTTATCGGGTCATGGCTGCGGCTGCTCTTCAAGAGATTCGCGGCAACGAGACGTCCAAGCAACTGCTTCAGCGACAACCACAGCAGTCCTTGCAGAATCATCAGATGCAATTTCGTCCCCAGCAGCAACAACCTCCTCTACAGCAACAAGAGCAGCAGCAACAACAGCAGCAACAACAGCAACAACAACAGCAGCAGCTTAATATGCAACAGCAGCAGCAACAAGTTCTGCAGTCTCCTCAGCCGTTGCCATTACCTGATGTTACTGGGCCCTTACTCCAACTTTCATCAAGCAGGCCACAATCTCCTATGCAGTTATCTACATTGCGGGCGTCATCAGGATATACGGACTCGGATGTGCACTTATCTCCTGCTTCCACTGCGTCAGGATCATTTCCTTTACAGAGTCTTCTGAGCAGGACTCAACAAGGCAGTGTACTCACCACTGAAGATACAAATCAGTTTTCAAATCTTCTCCGCAGCAATCAAAGCGCAATGCAACAAGCTACTATGCTACCTGGTTCCGCTATGGTAGGGCGGGACTCTCCTGTATCGAGTGCCTGGTATTCTATGCGGGATTCCACTACATCCGATTGCGCTCATCCAAGTTCCCGCATGGGGCGAGCAGACTCATCCCCCAGTTCTGCTCCCACATATGCTCTTTCATCCAATGAACCTTGTCAATCTGGTTTGACAGCCCTACCCATGCAGAATCCTACTTATGCAGTGTTTCGGGACAATTCACAGGAGCAAGATCAAGTACAGTCAGATCCTCGTAGTCATCTCCTTTTCGGTGTATCCATTGATCAAATGCCAAACGGAGCTGGGGGTCTGGGATCAAGGGGCTTTGGTAAGGCGAAGGATAACCAGGCGCGATTCGCTGGCGGTAGCCTACTGCCTGCTCCTTATTGCTCATCGGCGGGACAAGATCTGCCCATCAGCCCTGGCATAATATCCCATGGTAGTATAAATGATAGCCAATTTATGCAGCGGGGATTCATGGCACCCGTATCTTCTCCCCAGCGGAGCTACACAAAGGTCTACAAGCTTGGTTCCGTCGGGAGATCCCTAGATGTGGCCCAGTTCACCAACTATACAGATTTGCGTGTTCACCTTGCCCGCATGTTCGGACTCGAGGGGCAGCTTGAAGATCCACAAAGATCAGGCTGGCAGCTCGTGTTCGTGGACAATGAGCAAGATGTGCTTCTAGTTGGCGACGACCCCTGGGATGAGTTCGTCAATTGCGTTCGTAGCATCCGGATCTTGTCACCGTCGGAAGTGATGCACATGAGCCAGGAAGGATTGGAGCTCCTGAATATCGTTCCGCCAACTGCCCCCCGGCCAACGAGCAGTGGTTCTGAGGATGGTGCCACCATGCCTGCCGGATTCGAGAAATCCTGTGGGAATGATGCCCAGCGGGGGTGCCCCTGA

**>MpARF2**

ATGTCAGAAGCATCTTCCATCACTCGTCACCCCTACAAGGCAAACACCGGGCCCCTGCTGAAGTTCCAGCAATCGTCGGATGCTTCATCCTTGCCGCCAATGGCACGTCCCATGGCGAGCAGACAGCTTGCTACATCTCACACCGCTGCCTCCAACGTCTCCGTAGCAGGCGATGATGGCATCGATGCCGAGCTGTGGTACGCCTGTGCTGGTCCTCAGAAGGCATTACCACCCGTAGGCAGCGTCGTGGCCTACTTGCCTCAAGGTCACATAGAGCAGGTCGCGTCTTTCAATAATCAAGAACTCGACGCCCAAATTCCTCGGTATAATCTGCCCGCAGTGATACCATGTATGCTCAACGACATACAACTCAGCGCGGATCCTGATTCCGACGAGGTCTATGCGACTCTCACCCTGTGTCCCATGAGCGAGCAACACGAAGACTCGTCCGACTGCGCCGAGCCCCCGCCACCTCCCAAGAGGAAGTCCCGCAGTTTTACCAAAACCCTCACTGTCTCTGATACCAGCACGCATGGAGGCTTCTCGGTGCCCCGACGCGCTGCCGATGACTGCTTGCCGAAATTGGATATGAGTCTCAACCCTCCAAATCAGGAATTAGTGGCCAAGGATCTTCATGGCAACGAATGGCGATTTCGTCACATATTTCGAGGTCAACCTAAACGCCACCTTCTCACTACGGGATGGAGCGTATTTGTGAGCCAAAAGAGGCTCGTCGCCGGAGACGCGGTGCTGTTTCTCAGGGGTGAGAATGGCCAGCTCAGAGTAGGAGTCCGGCGTGCTCCTCGCCAGCAACAACTGCAACCCAAAGTGTTGACGTCTCCGACGATGCACATCGGGGTTCTCGCGGCCGCTGCCCATGCGGCAACGGAGAAATCTCGATTCTCTCTAATTTACAACCCTCGATCTTGCCCTTCGGAGTTCGTTATTCCGTACTCTAAGTACCTCAAGGCTGTGAAAAGCAACTTCAACGTTGGCCAGCGTTTTAAAATGAAGTTCGAATCGGAGGACCCTTCAGATAGGAGGCATACGGGAACTATCACCGGAATTTGTGATTTCGACCCTGCCAGGTGGCCTGGCTCAGAGTGGAGATCCCTCCAGGTTAATTGGGACGAATCGTCCTCGAGCGAGAGACAGGAGAGGGTCTCACCATGGGAAGTGGAGCCCTTCTCTCCCTCCACGACCATCACTCCGTCGGTCAGTACTAGAAAGCGATTGAGGCCGGTCACCCAACCACATTCTGAGTCAGTGAACAGGAATGCCGTCGAGACAAGTAAGGCGCAAACTCAAACGATGAGGCTAGCGAGAGCTTTCCACGGCGGTCATGAAATGCTGCCGTCATCAGCCGAGGAGGAGGATGCGGAATCTCTTTCTGCCAAAATGTCTTGGATTAAGAGAGAGGACAACTTCAAGAGCGAAGCGCAGAGCGTTGGCTCTAGACAGGGCCCGGATAGTTGGATGTCTATCAGGAGACCCGACCCAGTGCAGGTCCCTGACATGTTTAGAAATCTCCCGGCTTCGGGAGTGCCGGATCTTCGAGGTATGATTGGCATCGAGAGGCGCCAGCAGGAACACTTAAAGTTCTGCGTCAAGCAATATCGGGAAAACAAGGATGAAATCTCGGGAACTACTCTGCAGCTGTCAAGTCCCCGACCTCCGAACTTGCAGAATTACGTCAAGTCATCCACAGATCTGAATCTCTCCGTGTCCTCGCCTGCGTCGAGCAACAAGGGATCGAGCCTTCTTTGGTCCAATTCTCAGTCTGTAACGTTGCCATCGTATAATGGTCACGAGTCGACGAACGCATCCTCTTGGCTCTCGTTTAGACCGGGACAGAGCGATGTTGCCGCCTCTTCGCCTCACTGCACTACATTGTCAATGAGCAATCTGCCTCCAGCTGATTCTGAAACGTCAAGCCATCCTTCGACTCCGAAGAGTTATCTATGGGAGAAGAGGATAAGGATGGAGCCGGACACGAACCGAGCAGCTGCTCCCGTTCAGAGTGAGCAAAAATGTAAAATTTTCGGGGTGCCTCTCGACAAGCCAACTCCGATCGTGATTCCTTCTCAAGTGCCAGGATCTAAGGCAGTGAGGAGCACGGACGATGGATCAGGACCGAGCAGCAGTCGTGGGCTGGAGAAAGTCGTGTCTCCCAGTCCGACTTCTTCCGCAGTCGGAGGACAGGAGCAGGATAAGGGTCCCCAGAGATCCAATAAGACGTCGCAAAATTTCCAACAAGGTCCAGTTCGTAGCTACACGAAGATTCACAAGCAGGGATCGTTCGGAAGATCGATCGACGTCCAAAGTTATGACGGCTACACCGACTTGTTAAGAAAGGTAGAAAACATGTTCGAGCTGAACGGAGAGCTGTTCGACAAGAAGTCCGGGTGGCAGCTCGTGTACACAGACCACGAAGACGACGTCCTCCTCGTGGGAGACGATCCCTGGATGGAGTTTGTGAGTTGCGTGAGGACGCTGAGACTGCTGAGCCCGGGCGAGGCGTCGTCGTCGGGCAAGAGCGGGCAGTCGCACGACGAGGACGCGGGCGCGGGCAAGGACGGGGCCAAGCGGTGCGACTCGTCCTCGCCGTCGGCGGGGGCGCGCGGCGACGACATGTAG

**>MpARF3**

ATGCCCGGGCCAAGCCCTGGGTGTGGGACAATGAGCGGAACGAATATCAAGATGGAGAAATCGGAGGAGTCGATGGGAGGAGGAGGCAAGGGCTGGGGCGGGGGCAGAGATCGAGATTCATCGTCGGACGGAGGAGGGGGCTCGGGCGAGAACACGGGCCTGGATCCGCAGCTGTGGCACGCCTGCGCAGGAGGCATGGTGCAGCTGCCGCCCGTGGGCGCCAAGGTCATCTACTTCCCGCAGGGCCATGGAGAGCAGGCGGCCACGCCGCCCGAATTCCCAAGAATGATGGGCCCGCAGGGAACAATTGGGTGCCGAGTGGTGTCGGTGAGCTTCTTGGCCGACACGGAGACGGACGAGGTGTATGCGCGCATCCGCCTGCAGCCGCTGGAGCGCGAGGCCGCCATGTCCATCGCCGACTCGACGCTCGACGCGGACGGGGGCCCGTCGTCGCCCCCGCCCGAGAAGCCGGCCTCGTTCGCCAAGACGCTCACGCAGAGCGACGCCAACAACGGAGGCGGATTCTCGGTGCCGCGCTATTGCGCCGAGACCATCTTCCCGCCGCTCGATTACTCCATCGATCCGCCCGTGCAGACGGTGCTGGCCAAGGACGTGCACGGCGAGCGATGGAAGTTCCGCCACATCTACCGCGGCACTCCGCGCCGGCACCTGCTCACCACCGGCTGGAGCACCTTCGTGAATCAGAAGAAGCTCGTGGCCGGCGACGCCATTGTGTTTCTGCGCACCGCCAGCGGCGAGCTCTGCGTCGGCGTGCGCAGGTCGATGCGTGGCACGGGCGGCGCCGATTCCTCGACCTGGTCCGGCGGATCGTCGACCTCGCACCATCGCCCCAATCGCTGGGAGGTCAAGGGCACCGAGAGCTTCTCGGACTTCCTCGGCAACGACAGCGCTGCCGGAGGCGGCTCGGTCTCGAGCGCAGGCTCCGCTGCAGGCCCCGGAGGCCCTCGCGCTGGCCCCGGCGGCAGCAACTCGGGCCCCGGGATTGGCATCCCCGGCCCTTCCACCACCAGCAGCTTCGCGCGCAATCGAGCTCGCGTCACCGCGCAGTCGGTGCTCGAGGCCGCGTCGCTGGCCGTGCAGGGCCAGCCCTTCGAGGTCGTCTACTACCCTCGCGCCAGCACGGCCGAGTTCTGCGTCAAAGCGCAGGCCGTCAAGGCGGCCCTCGATCACACCTGGTTCCCGGGCATGCGCTTCAAGATGGCCTTCGAGACGGAGGATTCGTCGCGGATCAGCTGGTTCATGGGCACCATCTCCGCGGTGCAGCCCGCCGACTCGCTCTGGCCCAAGTCTCCGTGGCGCGTGCTTCAGGTGACATGGGACGAGCCGGATTTGTTACAGGGAGTGAGCCGCGTGAGTCCCTGGCAGGTGGAGTTGGTGTCGACTCTGCCGATGCAATTGCCTCCGTTCTCGCTGCCGAAGAAGAAGTTGAGGGCGGCTCAGCCCTCGGACATGAATATGCAAGGCCAAGGGCTGATGGGCATGCCCATCACACTACCTTCGGTTTTTGGGCAAATCAACCCCTGGGCGCACGGACTTACCATGGAGGAAGTTACTGCGGGCATGCAGGGAGCCAGGCATGATCGAGTTTTTGGGCTTGCCTTGTCGGAGAAATTCCGGCCTGGTAAACTCCCAGGTGGGTTTTTTTCAGCAGCAGAGGGTTATTATCCGGATCATGCTGGACGAGGTGGCGGTGGGGAGCCTCACCTTAGTGCATTCCCGCTCCAGGATCGTGCGAGCAACATTTCTTCCCTGATCAGCTCGCTGGGCAGCGTTCCGCCCAGTGGTGACCACGGATCGGCCGGCGCCTTGCTGGTCCCGGCCGGGTCCTGGTCCGGGGGCTCTGCGAACAACAAGAGTGCTTCCACTCAGCTGGTGCTGTTCGGCCAAGCAATCAACACCGACTCCAACAAGAGCCAGCCCCAACACTCAGGCGGGAGCTCGTGCGACGGCCCCTCGCTCCAACACTTGAAAGAGGAATCGTCCGGCAAGCGCAAGGATTCGCCTTCCGAATCCAACCAGAATGAGAATTTCGAGAGGGGCCAGAAGTACATGTCTGGCAGTCTGAGCAACGGCAAACAGTCGGATCTCATAGTGGGCGAGTTTCAGAAGTGGGTGCCCGGGTTGGACAAGGAGAGAGGTGCCGGAGAGAAGCTCGCCGCATCCCCTGAAATTTTGCAAAGTCCGCAAGGCGCCTGGTGA

**>MpTPL**

ATGTCATCGTTAAGCAGGGAGCTCGTGTTTCTGATACTTCAGTTCCTCGATGAGGAGAAATTCAAAGAAACCGTCCACAAGCTCGAGCAGGAGTCGGGCTTCTTCTTCAACATGAAGTACTTCGAGGACCAGGTCCAGGGCGGTGAATGGGAGGAGGTCGAACGCTACTTGTCTGGGTTCACCAAAGTCGATGATAACCGTTACTCCATGAAGATCTTTTTCGAGATCCGCAAACAGAAGTACCTGGAGGCTCTGGACAAGCAAGATCGAGCGAAGGCTGTGGATATTCTGGTGAAAGACTTGAAAGTTTTCTCCTCTTTCAACGAAGAACTGTACAAGGAGATTACCCAGCTTCTCACTCTGGAGAACTTTCGGGAGAACGAACAGTTGTCTAAGTACGGAGACACAAAGTCGGCAAGAAACATCATGCTCATCGAGTTGAAGAAGCTGATTGAAGCAAATCCGTTGTTCCGTGACAAGCTCACATTCCCGGCCCTGAAGGCGTCCCGATTGAGGACCCTTATCAATCAAAGCCTGAATTGGCAGCACCAGTTGTGCAAAAATCCCAGGCCGAATCCTGACATCAAGACTCTTTTCATCGACCACACATGCGGCCCTCCGAATGGCGCACGCGCTCCACCACCTGCCAACAACCCACTTGTTGGCACTCTGCCTAAGGGAGGAACATTCCCACCGATTGGTAGTCATAATTCCGTTCAGCTGCAGCCTTTCCAGCCAACCCCAGCGCCTTCCGCTAGTGCACTGGCCGGTTGGATGGCCAATCCCAACACCGCCGCTCCCCACGCCGCCGTTCCAGCTGGACCAGCTGCTCTTACTCCCTCTCCGAATCCAGCTTTATTGAAGAGGCCTCGCACTCCACCATCAAACGCTCCTGCAGTGGATTATCAGTCTGCCGATTCTGATCATCTCATGAAACGACCACGACCTGGTGGCCAGCCAGTTGAGGAGGTGAAAGCGCAAGTGTACAATGCTGGTGGTCCCAGCCACCCACAAAATGCTTACACTCCAGACGATCTTCCCAAGAATGTTGCCAGGACTCTTCATCAGGGCTCTTGTGTTATGAGCATGGATTTCCATCCCGTCCAACAGACAGTATTGCTAGTTGGAACGAATGTTGGCGATATTGGTATCTGGGAAGTTGGATCGAGAGATAGGCTTGCGCAGAGGACATTCAAAGTTTGGGACCTTTCAGTTTGTGGTATGCCTTTACAAGCTGCTCTTGTCAAAGATCCGGCTGTTGCTGTCAATCGGACTGTGTGGAGTCCAGATGGAAGTTTGCTCGGTGTTGCATTTTCCAAGTACATTGTCCATATCTACGCCTACAACGGAGGGAGCGATTTGAGGCAACATTTGGAGATTGACGCACATGTTGGCGGGGTGAATGATCTTGCGTTCTCTCATCCTAACAAACAACTCTGCGTCATCACATGTGGTGATGACAAAACCATAAAGGTGTGGGATGCAGTCACTGGGCGAAAGAATTACACCTTTGAAGGGCATGAGGCTCCTGTGTACTCTGTGTGTCCTCATCACAAAGAGAGCATTCAGTTTATCTTCTCCACTGCTATCGATGGAAAAATCAAGGCGTGGTTGTATGATCTCTTGGGATCTCGAGTGGACTACGATGCTCCTGGCCATTGGTGTACAACAATGGCGTACAGCGCCGACGGAACAAGATTGTTCTCTTGCGGTACAAGTAAAGAGGGTGAGTCTTATCTTGTCGAATGGAATGAGAGCGAAGGAGCCATCAAGCGAACGTACTCTGGGTTCCGCAAGAGATCGTTGGGTGTTGTGCAGTTTGACACTACACGGAATCGTTTTCTGGCTGCTGGCGATGAGTTTCTCATCAAATTTTGGGATATGGATAACGTCAACCTTTTGACTACTATTGAGGCTGATGGTGGTCTGCCTGCCAGTCCAAGGCTACGGTTCAACAAAGAGGGATCACTGCTGGCTGTGACTTCCGCTGATAATGGCATCAAGATTCTTGCGAACTCTGATGGAATGAGGCTGTTGAGAGCATTCGAGAGCAGAGCCTTCGAGAATACCCGAGGACCTCCAGAGCCTGCTGTTACCAAGGCACCTGGAGTAAACTCTATGACGGCGGTTAGTAGTGTCCCTTCCGGACCTCCTGTTGGAGGTCCGGATCGACCCGACAGAAATACTGCCTCGGCCACAATGGCGGGTCCATCGATGGACGGGTCAAATCTGATTGTGGGCCAGGCGCGTCCCCGGGACAGGGGTGGAAATGACCATGTGACCCTTTCAAATCCGACCAATTTTTCACGAAACGCCGACGGGAATGGAGTCGACAACAATCGTCCTCCAGAACAAAAACCAAGGGTACCAGAAGACTTGACGGACAGAGTTAAGAGTTGGAAGTTGACTGAGATATCTGATCCAGGTCAATGTAGGTCGACTAGACTTCCTGACACTTTACCTGCAAGCAAGATATCGAGGTTGATTTACACCAACTCCGGAGTGGCACTGCTAGCTCTTTGCTCGAGTGCCGTTCATAAGCTATGGAAGTGGCAACGCACTGAGCGTAATCTTACTGGAAAGGCCACGGCAACTGTACCACCCCAACTCTGGCAACCAGCGAGTGGTATTCTTATGACCAACGATATAAGCGACAACTCTTCAAATCCGGAGGAAGCAGTACCCTGTATTGCGCTTTCAAAGAATGATTCGTATGTCATGTCAGCATCTGGAGGAAAAGTGTCATTGTTCAACATGATGACCTTTAAGGTTATGACCACATTTATGCCGCCACCACCTGCGGCAACTTTTCTTGCGTTCCATCCTCAAGACAACAACATTATTGCTATAGGGATGGAGGATTCAACTATACAGATTTACAACGTCAGGGTAGACGAGGTAAAGAGTAAACTAAAAGGACATCAGAAGAGAATTACAGGATTGGCATTTTCAAATGCTTTGAATGTTTTAGTTTCTTCTGGTGCAGACGCACAGTTGTGTGTATGGGGTACGGATGGTTGGGAGAAGCGAAAGGCGAAATTTATCCAAACAGGACGTGCACCCCAACCTGTGGGTGACACCAGAGTACAGTTTCATAATGATCAGATCAGGCTACTTGTAGTACATGAGACACAACTCGCAATATACGACGCTTCGAAGTTGGAACGCATTCGTTATTGGGTACCGCGAGATTCTTTCACAGCTTCAATTTCAAATGCAACTTATTCCTGCGATAGTCAGTTGGTATATGCGGGATTCGTAGATGGATCCATCGGTGTTTTTGACGCGGAGAGTCTACGTCCACGTTGTCGTTTGGCGGCATCGGTCCACATTCCTCCTGGTGTTAGCGGAGCTACTGCATATCCTCTTGTGATCGCAGCACATCCTTCTGAAGCCAACCAGTTTGCGCTGGGGTTGAGTGATGGGGCTGTTCAGGTTATCGAACCTTTGGAATCAGAGGGAAAGTGGGGCATTGGTCCTCCCTCCGATAATGGAGGACCTTCAAGCGTGTCTGGACAAACCTCCATTCAAAATTCTGATCAAGCTCCGAGATGA

**FIGURE S13. Sequences used in this study.** *miR160* binding site of *MpARF3* is indicated in red.
