# Supplementary material for: A Simple Auxin Transcriptional Response System Regulates Multiple Morphogenetic Processes in the Liverwort Marchantia polymorpha
Source: PLoS Genet. 2015 May 28;11(5):e1005207. doi: 10.1371/journal.pgen.1005207 (PMC4447368; doi:10.1371/journal.pgen.1005207)
Supplement: Supplementary file 3 [file pgen.1005207.s014.doc]

Table S1. D34 protein sequence identity based on EMBOSS Matcher Pairwise alignment

**Identity %**	MpIAA	MpARF1	MpARF2	MpARF3	AtBDL	AtARF10
MpIAA	1					
MpARF1	**0.39**	1				
MpARF2	**0.37**	0.61	1			
MpARF3	**0.39**	**0.35**	**0.31**	1		
AtBDL	0.66	0.42	0.41	0.41	1	
AtARF10	0.40	**0.33**	**0.32**	0.47	**0.38**	1
