# Supplementary material for: A Simple Auxin Transcriptional Response System Regulates Multiple Morphogenetic Processes in the Liverwort Marchantia polymorpha
Source: PLoS Genet. 2015 May 28;11(5):e1005207. doi: 10.1371/journal.pgen.1005207 (PMC4447368; doi:10.1371/journal.pgen.1005207)
Supplement: Supplementary file 4 [file pgen.1005207.s015.doc]

Table S2. $_{pro}$*EF1:MpTPL*^{N176H} results in additional branching events.

Phenotype	Notches (25 days of growth)	Std. Deviation	two tailed t-test (P value)
HART (N=17)	11.29411765	3.312187832	2.56844E-12
$_{pro}$*EF1:MpTPL*^{N176H} (N=17)	26.35294118	4.372373161	
