# Supplementary material for: A Simple Auxin Transcriptional Response System Regulates Multiple Morphogenetic Processes in the Liverwort Marchantia polymorpha
Source: PLoS Genet. 2015 May 28;11(5):e1005207. doi: 10.1371/journal.pgen.1005207 (PMC4447368; doi:10.1371/journal.pgen.1005207)
Supplement: Supplementary file 5 [file pgen.1005207.s016.doc]

Table S3. Notch and gemmae cup production in mature *MpARF1* overexpressing lines (N=30)

Genotype	Cups	Std. Deviation	Std. Error	Notches	Std. Deviation	Std. Error	Cup/Notch	Std. Deviation	Std. Error
TATA:GUS	10.8667	4.288986162	0.783058	19.52222	5.812974874	1.061299	0.5720936	0.200845615	0.036669
EF1:MpARF1	0.05882	0.242535625	0.058824	26.52941	7.633132115	1.851306	0.0018382	0.007579238	0.001838
EF1:MpARF1ΔD34	11.7549	7.395539116	1.268324	33.51471	9.87722337	1.69393	0.3710631	0.220662061	0.037843
