# Supplementary material for: A Simple Auxin Transcriptional Response System Regulates Multiple Morphogenetic Processes in the Liverwort Marchantia polymorpha
Source: PLoS Genet. 2015 May 28;11(5):e1005207. doi: 10.1371/journal.pgen.1005207 (PMC4447368; doi:10.1371/journal.pgen.1005207)
Supplement: Supplementary file 6 [file pgen.1005207.s017.doc]

**Table S4. Summary of phenotypic effects of constructs tried in this work.**		
Putative effect on auxin signaling	Genotype / Treatment	Phenotype
**Loss of auxin signaling**	L-Kyn treatment (100 µM)	Delays in cup differentiation, delays in branch separation, gemma cup fusion, compromised rhizoid production and delayed growth.
	proEF1:MpTPL	Loss of gemmae cup differentiation.
	MpTPL-D34$^{IAA, ARF1, ARF2}$	Severe loss of growth, patterning, and 3D growth
	$_{pro}$*EF1:iaaL / proSHI:iaaL*	Severe loss of growth, patterning, and 3D growth
	$_{pro}$*EF1:MpIAA-DII* (Kato et al 2015)	Severe loss of growth, patterning, and 3D growth
	CRISPR-CAS *Mparf1*- alleles (Sugano et al, 2014).	Diminished area / auxin resistance / loss of gemmaling dormancy
**Over-active auxin signaling**	Exogenous 2,4-D, NAA (10 µM)	Ectopic rhizoids, elongated cups, branching suppression, growth suppression.
	$_{pro}$*EF1:amiRMpIAA*$_{no7/no9}$	Auxin hypersensitivity – ectopic formation of rhizoids to low auxin concentrations
	$_{pro}$*EF1:MpARF1*	Extra bifurcations
	$_{pro}$*EF1:MpARF1ΔD34*	Extra bifurcations / longer rhizoids
	$_{pro}$*EF1:MpTPL*^{N176H}	Extra bifurcations / elongated gemma cups
