# Supplementary material for: A Simple Auxin Transcriptional Response System Regulates Multiple Morphogenetic Processes in the Liverwort Marchantia polymorpha
Source: PLoS Genet. 2015 May 28;11(5):e1005207. doi: 10.1371/journal.pgen.1005207 (PMC4447368; doi:10.1371/journal.pgen.1005207)
Supplement: Supplementary file 7 [file pgen.1005207.s018.doc]

Table S5. Primers used in this study.

Primer Name	Sequence (5' to 3')
TPL-F1	GTNGAYGACAAYCGCTAYTCMATG
TPL-F2	CTKATYAAYCARAGYCTCAATTGG
TPL-R1	ATWGCNCCTTCRCTYTCATTCCT
TPL-R2	TCCCAMACCTTGATNGTYTTRTCATC
MpTPLF3	AGCTGCTCTTACTCCCTCTCCGAATCCAGC
MpTPLF4	GAATGTTGCCAGGACTCTTCATCAGGGCTC
MpTPLR3	AAGCTGGATTCGGAGAGGGAGTAAGAGCAG
MpTPLR4	GAGCCCTGATGAAGAGTCCTGGCAACATTC
MpTPL-SalI-F	ATGTCGACAGACAGGGGCGATAGGAACAGCGTACAATG
MpTPL-EagI-R	ATCGGCCGTTGTATACACTGGCTTTCAAGCAGCCACGG
MpTPL(N176H)-F	GGACCCTTATCCACCAAAGCCTGAATTGG
MpTPL(N176H)-R	CCAATTCAGGCTTTCGTGGATAAGGGTCC
MpEF1-NdeI-F	ATCATATGCAAATGAGTCACACACATTGTTGAG
MpEF1-SalI-R	ATGTCGACCAACCTTTCTGCAGGCACATCAATACTG
MpTPL pro F5-NdeI	ATCATATGTCTTGTTCCGGGAAATACCGGGATATAGTCC
MpTPL pro R1-SalI	ATGTCGACTGTACGCTGTTCCTATCGCCCCTGTCTATCGAATGG
MpTPL pro F1 int	TCGTGCGTGCGCGCTTCGGGTCGGTGCGCTTCTCC
MpTPL pro R1	TGTACGCTGTTCCTATCGCCCCTGTCTATCGAATGG
MpTPL pro F2-CORR	AGAACATTCAACGCGCGACGACTTCCTCAGCC
MpTPL pro F3-COR	ATCACCTGGTTCCAGTGCCGGGTAGTTAACC
MpTPL pro F4-COR	ACTTGAACACGAACCGGCTAGTTTTGCAACC
MpTPL pro F5-COR	TCTTGTTCCGGGAAATACCGGGATATAGTCC
EcoRI-MpIAA-DIII/IV-F	taGAATTCGCTGTTCAGTCAAACTCCTGGTTTGTG
HindIII-MpIAA-DIII/IV-R	taAAGCTTTCACACGTTCGGTTGAGTCGTCTTGTTTG
EcoRI-MpARF1-DIII/IV-F	TAGAATTCCCCGTATCTTCTCCCCAGCGGAGC
EcoRI-MpARF1-DIII/IV-R	TAGAATTCTCAGGGGCACCCCCGCTGGGCATC
EcoRI-MpARF2-DIII/IV-F	GAATTCTTCCAACAAGGTCCAGTTCGTAGCTACACG
HindIII-MpARF2-DIII/IV-R	ATAAGCTTCTACATGTCGTCGCCGCGCGCCCCGCC
MpARF3-D34REAL-EcoRI	AGAATTCGGTGAGTCCTTCTCGCACTGCAAAG
MpARF3-D34REAL-HindIII	AAAGCTTCTACTGCGACCGCGTTTTGCCGTTG
ME1	catatgAGGGGCACTTTTGCTGCATTGTGCT
ME2	ggtaccTTCCGCCCTCCACCGCCTGGCCCT
ME83	ggtaccATGACTGCCTACGATATGGAAAAGGAAT
ME84	aagcttTCAGTTTCGGCGGTCGATGATGTAGAGCA
MpARF(miR167)R3in	CAAGAACACCAATATGCATGCTGTCACTCC
MpARF(miR167)R4out	CCATCCTGAAACGCATCCCGACAGAAACC
MpARF(miR167)F1	TTCGGGTGAAGATGAGGAAGGAGATGG
MpARF1(BamHI)F1	ATGGATCCAGGCTGATTGAGGTTGGCCGTCTGGGTC
MpARF1(EcoRV)R1	ATGATATCCCTTTTAGCCAAAGAATGTATTGAGCTCAC
MpARF1-atg-F-KpnI	atGGTACCATGTATTCTTGTTCGCCGATGAGGCTG
MpARF1-DeltaD34-R-HindIII	atAAGCTTTCACATGAATCCCCGCTGCATAAATTG
MpARF1-mNotI-F2	CAGCCGCTAACCATGCAGCCGCCACCAATAG
MpARF1-mNotI-R2	CTATTGGTGGCGGCTGCATGGTTAGCGGCTG
MpARF1.STP.BamHI-R	atGGATCCTCAGGGGCACCCCCGCTGGGCATCATTC
RLM-MpIAA-R1	AGTTCTTCCGGAACGATCGAATGGGAGGCC
RLM-MpIAA-R3	AGGAAATTGCGCTTAATATCGGAGGCGAGG
