# Supplementary figures and images for: Correction: A Simple Auxin Transcriptional Response System Regulates Multiple Morphogenetic Processes in the Liverwort Marchantia polymorpha
Source: PLoS Genet. 2016 Feb 22;12(2):e1005900. doi: 10.1371/journal.pgen.1005900 (PMC4764325; doi:10.1371/journal.pgen.1005900)

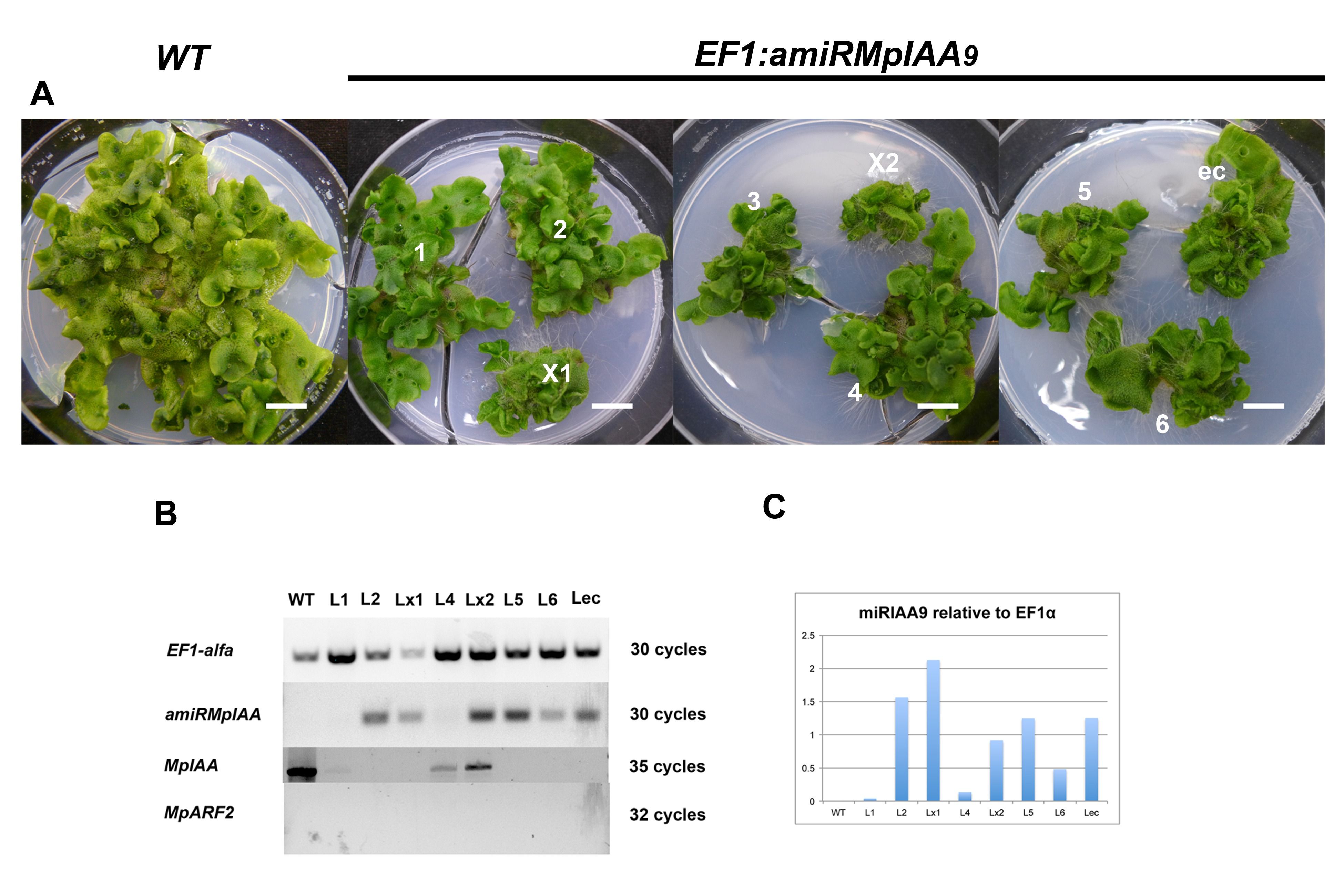

Supplement: S8 Fig — (A) Phenotypes of several independent lines constitutively expressing amiRMpIAA9. (B) Semi-quantitative RT-PCR showing transgene (amiRMpIAA9) and full-length target (MpIAA) levels in thallus tissues. (C) Transcript levels relative to EF1-alfa control. Lines with the weakest phenotype (line 1 and 4 as seen in A) have the lowest amiR transgene levels. (JPG) [file pgen.1005900.s001.jpg]
